# VANGL2 downregulates HINT1 to inhibit the ATM-p53 pathway and promote cisplatin resistance in small cell lung cancer

**DOI:** 10.1038/s41420-025-02424-w

**Published:** 2025-04-08

**Authors:** Jiayi Xie, Huiying Liu, Chunqian Yang, Weitao Shen, Jian Zhang

**Affiliations:** 1 Department of Oncology, Zhujiang Hospital, Southern Medical University, Guangzhou, China; 2 School of Basic Medical Sciences, Chongqing Medical University, Chongqing, China

**Keywords:** Small-cell lung cancer, Chemotherapy

## Abstract

Cisplatin is a first-line drug for the treatment of small cell lung cancer (SCLC). Although the majority of patients with SCLC initially respond to cisplatin therapy, cisplatin resistance readily develops, leading to tumor progression. Therefore, this study aims to elucidate the mechanisms underlying cisplatin resistance in SCLC. We found that VANGL2 is a poor prognostic factor and promotes cisplatin resistance in SCLC. Mechanistically, in cisplatin-resistant cells, VANGL2 overexpression leads to the autophagic degradation of HINT1. This reduction in HINT1 expression further reduces the phosphorylation of ATM and p53 induced by cisplatin-mediated DNA damage. The decreased phosphorylation of p53 inhibits downstream apoptotic pathways, thereby reducing cisplatin-induced apoptosis. In conclusion, VANGL2 regulates the ATM-p53 pathway-mediated apoptotic response of SCLC to cisplatin by downregulating HINT1, thereby promoting cisplatin resistance. Thus, VANGL2 may serve as a potential therapeutic target for reversing cisplatin resistance in SCLC patients.

## Introduction

Among all cancer types worldwide, lung cancer has the highest morbidity and mortality rates [1]. Small cell lung cancer (SCLC), which accounts for approximately 13% of all newly diagnosed lung cancers, is considered the most aggressive subtype [2]. Platinum-containing chemotherapy, particularly cisplatin, has served as the cornerstone of SCLC treatment for the past several decades [3]. Although most SCLC patients achieve a good response to initial treatment with cisplatin, many of them develop cisplatin resistance and eventually die of disease recurrence [4, 5]. Currently, the mechanisms underlying cisplatin resistance in SCLC remain incompletely understood. Therefore, elucidation of the mechanisms of cisplatin resistance is essential for SCLC patients.

Vang-like protein 2 (VANGL2), a key player in the planar cell polarity pathway, significantly influences the development of cell and tissue polarity, growth, and has implications in cancer [6]. VANGL2 has been observed to be overexpressed in breast, ovarian, and endometrial malignancies [7] and has been shown to promote breast cancer invasion and metastasis [8]. In addition, VANGL2 can activate the JNK pathway, thereby promoting the proliferation of breast cancer cells [9]. However, there are no reports that VANGL2 is associated with cisplatin resistance, and it is not clear whether VANGL2 is involved in cisplatin resistance in SCLC.

Cisplatin is a first-line chemotherapeutic agent that induces DNA damage, and failure to repair DNA damage can lead to cancer apoptosis [10, 11]. When severe cisplatin-induced DNA damage occurs, ataxia telangiectasia mutated (ATM) is rapidly autophosphorylated and activated. ATM induces p53 Ser46 phosphorylation, which activates the apoptotic pathway and leads to the apoptosis of tumor cells [12, 13].

In this study, we found that VANGL2 was highly expressed in cisplatin-resistant SCLC cells and that VANGL2 overexpression was associated with poor prognosis in SCLC patients. We further found that VANGL2 could modulate HINT1 expression at the post-translational level. The phosphorylation response of ATM and P53 in response to cisplatin stimulation was found to be reduced by VANGL2, which regulated HINT1, thereby reducing DNA damage-induced apoptosis and promoting cisplatin resistance in SCLC. In conclusion, our findings suggest that VANGL2 may be a therapeutic target for reversing cisplatin resistance in SCLC.

## Results

### VANGL2 is highly expressed in chemoresistant SCLC cell lines and is associated with poor patient prognosis

**Fig. 1:**
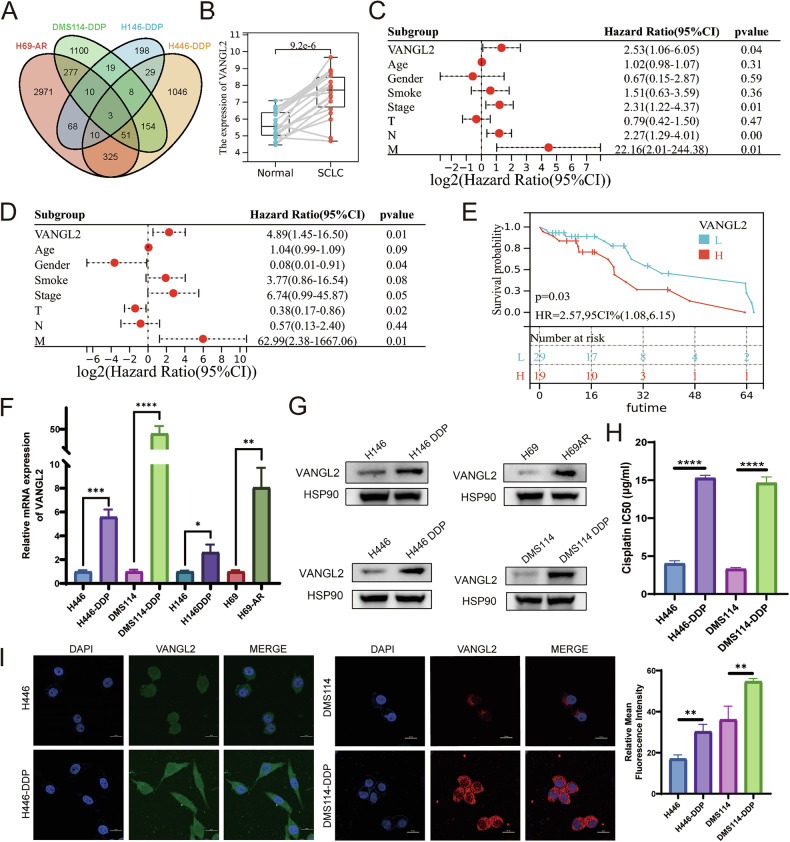
VANGL2 expression and clinical significance in SCLC. **A** Venn diagram of the intersection results of SCLC chemoresistance-related genes. **B** The mRNA expression levels of VANGL2 between SCLC and adjacent normal tissues. **C**, **D** Univariate Cox analysis (**C**) and multivariate Cox analysis (**D**) of VANGL2 expression and clinicopathologic characteristics in SCLC patients. **E** Kaplan–Meier curves of OS comparing the high and low VANGL2 expression groups in GSE60052 SCLC cohort. **F** The qRT-PCR analysis of VANGL2 mRNA expression in four pairs of chemosensitive and chemoresistant SCLC cells. ^*^P < 0.05, ^**^P < 0.01, ^***^P < 0.001, ^****^P < 0.0001. **G** Western blot analysis of VANGL2 expression in four pairs of chemosensitive and chemoresistant SCLC cells. **H** CCK8 assays showed the cisplatin IC50 of cisplatin-sensitive cells and cisplatin-resistant cells (H446/H446-DDP and DMS114/DMS114-DDP). ^****^P < 0.0001. **I** Immunofluorescence analysis of VANGL2 expression in H446/H446DDP and DMS114/DMS114DDP cells. Scale bars = 20 µm. ^**^P < 0.01.

Our laboratory used high-throughput whole-transcriptome sequencing of four pairs of chemoresistant and paired chemosensitive SCLC cell lines (DMS114-DDP/DMS114; H146-DDP/H146; H446-DDP/H446; and H69-AR/H69) to perform differential expression analysis (DEA). We overlapped the 4 sets of genes upregulated in the chemoresistant cells (screening conditions were logFC > 1 and *P* < 0.05). VANGL2, SYT2, and PLSCR4 were found to be upregulated in four chemoresistant SCLC cell lines compared with chemosensitive SCLC cells (Fig. 1A). The expression of these three genes in SCLC tissues was analyzed using the GSE149507 database, which consists of RNA sequencing data from 18 SCLC tissues and paired adjacent normal lung tissues. The results revealed that only VANGL2 expression was significantly higher in SCLC tissues than in adjacent normal lung tissues (Figs. 1B and S1A). Therefore, we selected the VANGL2 gene for further analysis.

We further analyzed the relationship between VANGL2 expression and overall survival (OS) in SCLC patients using the GSE60052 cohort. Univariate and multivariate Cox regression analyses of OS revealed that VANGL2 was an independent prognostic factor for the survival of SCLC patients (Fig. 1C, D). Survival curves revealed that high VANGL2 expression was associated with shorter OS in SCLC patients and that VANGL2 was a poor prognostic marker for SCLC patients (Fig. 1E).

To further clarify whether VANGL2 plays a key role in SCLC chemoresistance, qRT-PCR and Western blotting were used to analyze VANGL2 expression. We found that VANGL2 mRNA and protein expression levels were significantly higher in chemoresistant cell lines than in their chemosensitive counterparts (Fig. 1F, G), validating the transcriptome sequencing results.

Since VANGL2 expression was significantly upregulated at both the RNA and protein levels in H446-DDP and DMS114-DDP cells, which were cisplatin-resistant cells constructed by continuous incubation with cisplatin (Fig. 1H). Therefore, these two pairs of cisplatin-resistant and cisplatin-sensitive SCLC cells (H446-DDP/H446 and DMS114-DDP/DMS114) were selected for further experimental studies. Immunofluorescence (IF) also revealed that VANGL2 was highly expressed in cisplatin-resistant SCLC cell lines and that it was localized in the cytoplasm and nucleus (Fig. 1I).

### VANGL2 promotes cisplatin resistance in SCLC in vitro

**Fig. 2:**
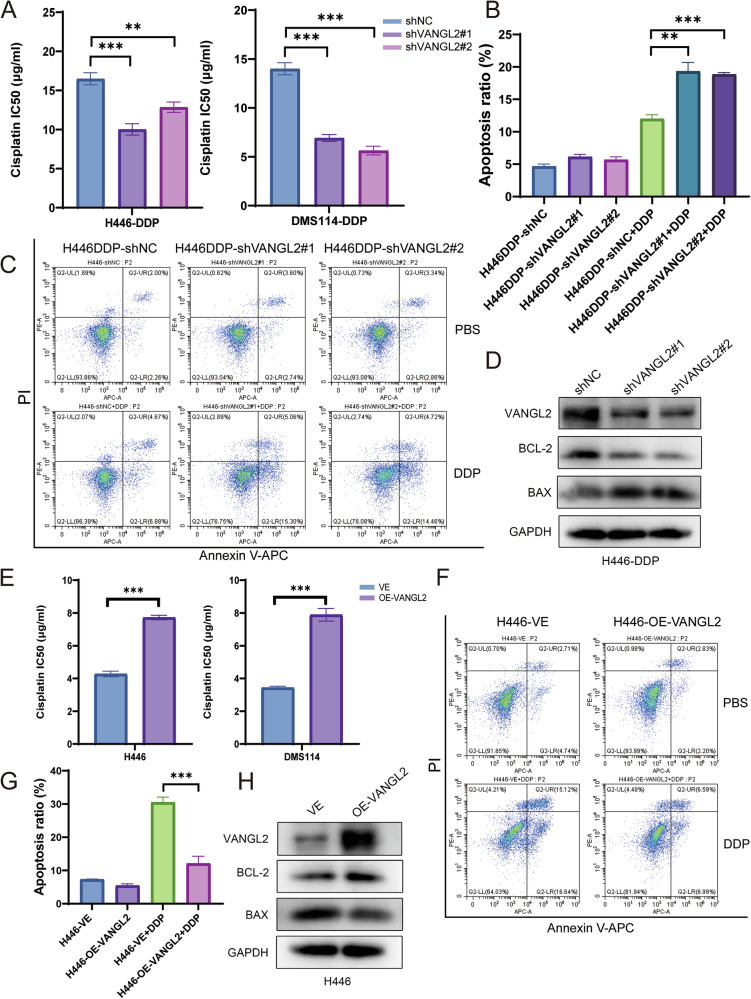
VANGL2 induced SCLC cisplatin resistance in vitro. **A** CCK-8 assays showed knockdown of VANGL2 reduced the cisplatin IC50 values in cisplatin-resistant H446-DDP and DMS114-DDP cells. ^**^P < 0.01,^***^P < 0.001. **B**, **C** Knockdown of VANGL2 resulted in an increase in cisplatin-induced apoptosis. ^**^P < 0.01, ^***^P < 0.001. **D** Apoptosis-related protein levels were measured by Western blot assays following treatment with cisplatin in VANGL2 knockdown cells. **E** CCK-8 assays showed overexpression of VANGL2 increased the cisplatin IC50 of ciplatin-sensitive H446 and DMS114 cells. ^***^P < 0.001. **F**, **G** Overexpression of VANGL2 resulted in the reduction of cisplatin-induced apoptosis. ^***^P < 0.001. **H** Apoptosis-related protein levels were measured by Western blot assays following treatment with cisplatin in VANGL2 overexpressing cells.

To further evaluate whether VANGL2 is involved in cisplatin resistance in SCLC, we established stable VANGL2-knockdown DMS114-DDP and H446-DDP cell lines by lentiviral transduction (Fig. S1B, C). The half maximal inhibitory concentration (IC50) and degree of cell apoptosis were assessed after 24 h of cisplatin treatment. We found that the knockdown of VANGL2 in cisplatin-resistant cells significantly reduced the cisplatin IC50 (Fig. 2A). The flow cytometry results also revealed that after treatment with cisplatin, the proportion of apoptotic cells in the VANGL2-knockdown group was significantly higher than that in the control group (Figs. 2B, C and S2A, B). Total protein was collected after 24 h of cisplatin treatment, and the protein levels of the proapoptotic protein BAX and the antiapoptotic protein BCL-2 were detected by Western blotting. Western blotting revealed that the expression of BAX was increased, while the expression of BCL-2 was decreased in VANGL2-knockdown cells treated with cisplatin, which was consistent with the results of the flow cytometry assays (Figs. 2D and S2C).

To complement our findings in cisplatin-sensitive cell lines, we overexpressed VANGL2 in H446 and DMS114 SCLC cells (Fig. S1D, E). As expected, VANGL2 overexpression increased the IC50 values of cisplatin in cisplatin-sensitive cells (Fig. 2E). Flow cytometry analysis revealed that VANGL2 overexpression significantly reduced the proportion of apoptotic cells, and the results of the Western blotting of apoptotic proteins were consistent with the previous results (Figs. 2F–H and S2A–C).

Collectively, these results indicated that VANGL2 expression promoted SCLC cisplatin resistance in vitro.

### VANGL2 induces cisplatin resistance in SCLC in vivo

**Fig. 3:**
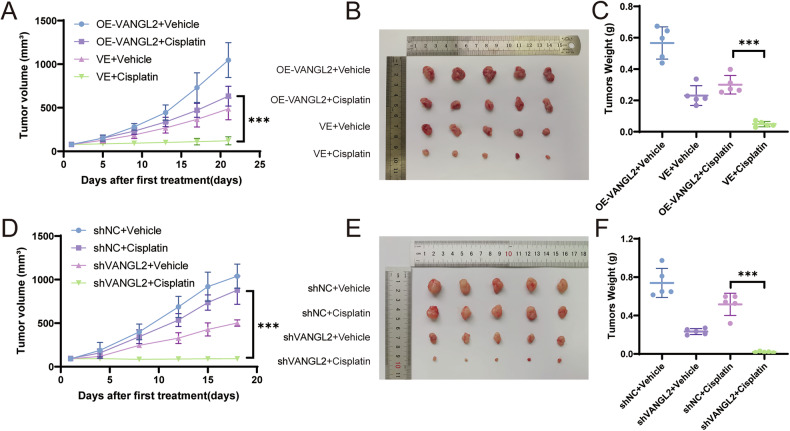
Effect of VANGL2 on SCLC cisplatin resistance in vivo. **A** Tumor growth was measured in the VANGL2-overexpressing and control groups treated with cisplatin or saline. ^***^P < 0.001. **B**, **C** Effect of VANGL2 overexpression on SCLC cisplatin resistance in nude mice. The tumor weights were measured. ^***^P < 0.001. **D** Tumor growth was measured in the VANGL2 down-regulated and control groups treated with cisplatin or saline. ^***^P < 0.001. **E**, **F** Effect of VANGL2 knockdown on SCLC cisplatin sensitivity in nude mice. The tumor weights were measured. ^***^P < 0.001.

To evaluate the role of VANGL2 in vivo, we established subcutaneous xenograft models using VANGL2-overexpressing H446 cells or VANGL2-knockdown H446-DDP cells. We subcutaneously injected cells with stable overexpression or knockdown of VANGL2 into nude mice, and the mice were intraperitoneally administered with cisplatin or saline. VANGL2 overexpression significantly accelerated the growth of the transplanted tumors (Fig. 3A) and significantly promoted the volume of the transplanted tumors after cisplatin treatment (Fig. 3B, C). In contrast, the growth rate and volume of the VANGL2-knockdown tumors were significantly lower than those of the negative control (NC) tumors (Fig. 3D–F). The results of these experiments indicated that VANGL2 promoted cisplatin resistance in SCLC in vivo and confirmed the efficacy of VANGL2 as an effective therapeutic target for reversing cisplatin resistance in SCLC.

### VANGL2 regulates the expression of HINT1

**Fig. 4:**
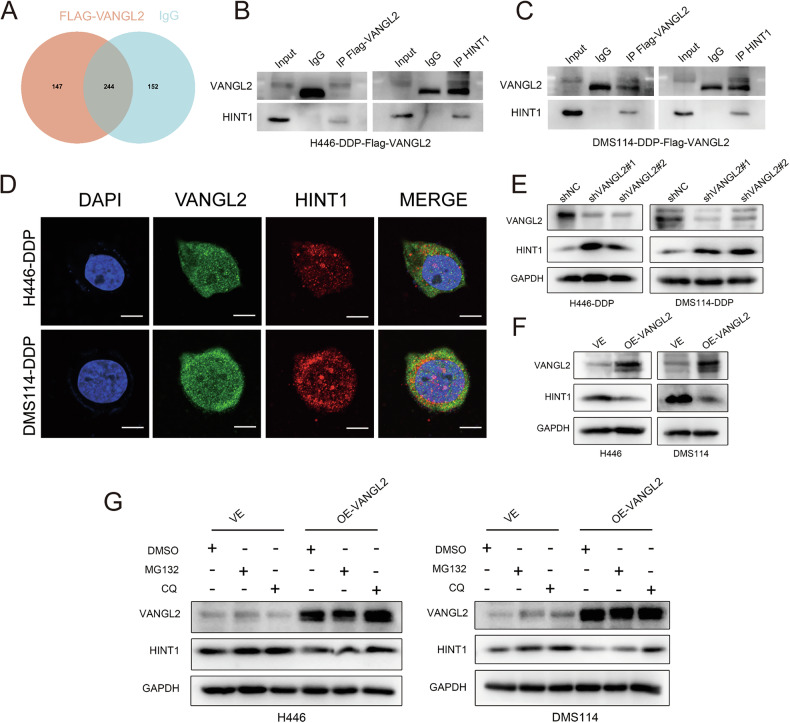
VANGL2 negatively regulated HINT1. **A** Venn diagram showed the results of mass spectrometry. **B**, **C** The co-immunoprecipitation experiments demonstrated the interaction between VANGL2 and HINT1. **D** IF analysis showed co-localization of VANGL2 with HINT1 in cisplatin-resistant SCLC cells. Scale bar = 10 µm. **E**, **F** Western blot assays detected the protein levels of HINT1 after knockdown or overexpression of VANGL2. **G** SCLC cells were treated with MG132, CQ, or DMSO for 12 h after overexpression of VANGL2 or control, and HINT1 protein expression levels were detected by Western blot assays.

To further clarify the mechanism of VANGL2 in SCLC cisplatin resistance, we stably transfected VANGL2-overexpressing lentivirus with a FLAG tag into cisplatin-resistant H446-DDP cells, and then performed immunoprecipitation experiments with FLAG antibody and control IgG antibody. Next, liquid chromatography-mass spectrometry(LC-MS) was used to analyze the potential proteins that might interact with VANGL2. After excluding non-specifically interacting proteins in the IgG group, 147 candidate interacting proteins remained, and the top 5 of these proteins were selected for analysis (Fig. 4A). Previous studies have shown that HINT1 is a tumor suppressor that inhibits tumor proliferation and induces apoptosis and that deletion of HINT1 promotes tumor resistance to radiotherapy [14]. However, the effect of HINT1 on cisplatin resistance in SCLC has not been reported, and the relationship between VANGL2 and HINT1 remains to be elucidated. We speculated that VANGL2 may affect cisplatin resistance in SCLC by interacting with HINT1. To further verify the relationship between HINT1 and VANGL2, we performed coimmunoprecipitation (Co-IP) assays using H446-DDP-Flag-VANGL2 and DMS114-DDP-Flag-VANGL2 cells (Fig. 4B, C), and the Co-IP assays confirmed binding between VANGL2 and HINT1. In addition, we performed IF experiments, and confocal microscopy revealed the colocalization of VANGL2 with HINT1 in cisplatin-resistant H446-DDP and DMS114-DDP cells (Fig. 4D).

To further investigate how VANGL2 interacts with HINT1, we examined whether the mRNA or protein levels of HINT1 were altered after knockdown or overexpression of VANGL2. Western blotting revealed that HINT1 protein expression was upregulated after VANGL2 knockdown in H446-DDP and DMS114-DDP cells (Fig. 4E). Consistently, the protein expression level of HINT1 was downregulated after VANGL2 overexpression in H446 and DMS114 cells (Fig. 4F). However, qRT-PCR revealed that the mRNA expression of HINT1 was not significantly altered regardless of whether VANGL2 was knocked down or overexpressed (Fig. S3A, B). In addition, the overexpression of HINT1 in H446-DDP and DMS114-DDP cells had no significant effect on VANGL2 protein or mRNA expression (Fig. S3C, D). These results confirmed that VANGL2 can negatively regulate HINT1 expression at the post-translational level.

Since the ubiquitin-proteasome pathway and the autophagy-lysosome pathway are the two major protein degradation pathways in eukaryotic cells, we further investigated the pathways by which VANGL2 regulates HINT1. We transfected VANGL2-overexpressing lentivirus or control lentivirus into H446 and DMS114 cells, added the proteasome inhibitor MG132, the autophagy inhibitor chloroquine, or the corresponding volume of DMSO, respectively, and collected total proteins after treatment for 12 h. Western blotting revealed that the autophagy inhibitor chloroquine could restore the downregulated protein expression of HINT1 affected by VANGL2 overexpression, whereas the proteasome inhibitor MG132 had no significant effect on the downregulation of HINT1 affected by VANGL2 overexpression (Fig. 4G). These results suggested that VANGL2 regulated HINT1 mainly through the autophagy-lysosome pathway but not through the ubiquitin-proteasome pathway.

### HINT1 affects SCLC cisplatin sensitivity by regulating cisplatin-induced apoptosis

**Fig. 5:**
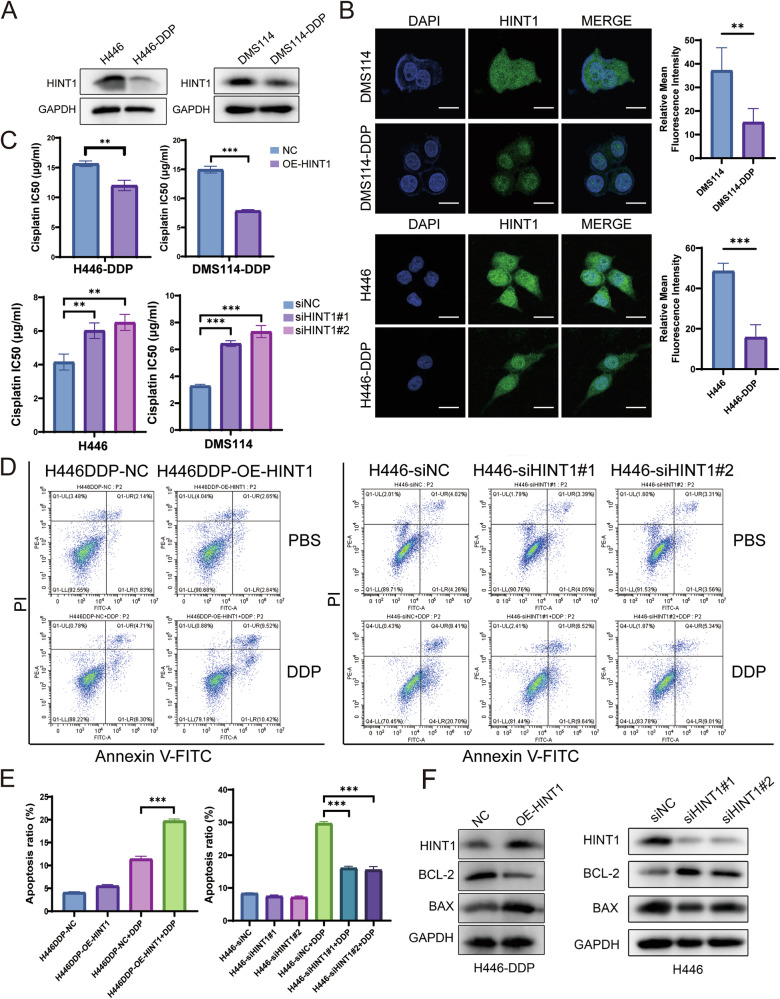
Downregulation of HINT1 promoted cisplatin resistance in SCLC. **A** Western blot assays showed HINT1 expression in cisplatin-sensitive and cisplatin-resistant SCLC cells. **B** IF analysis of HINT1 expression in two pairs of cisplatin-sensitive and cisplatin-resistant SCLC cells. Scale bar = 20 µm. ^**^P < 0.01, ^***^P < 0.001. **C** CCK-8 assays showed that the overexpression of HINT1 decreased the cisplatin IC50 values in cisplatin-resistant H446-DDP and DMS114-DDP cells, and the knockdown of HINT1 increased the cisplatin IC50 values in H446 and DMS114 cells. ^**^P < 0.01, ^***^P < 0.001. **D**, **E** Overexpression of HINT1 in cisplatin-resistant SCLC cells resulted in an increase in cisplatin-induced apoptosis, and knockdown of HINT1 in cisplatin-sensitive SCLC cells resulted in a decrease in cisplatin-induced apoptosis. **F** Apoptosis-related protein levels were measured by Western blot assays in HINT1-overexpressing or HINT1-knockdown cells following treatment with cisplatin.

To determine whether the regulation of HINT1 by VANGL2 contributes to cisplatin resistance in SCLC, we examined the relationship between HINT1 and cisplatin resistance in SCLC. Both Western blot and IF assays revealed that HINT1 expression was significantly lower in cisplatin-resistant cells than in paired cisplatin-sensitive cells (Fig. 5A, B). In addition, the HINT1 protein was expressed in the cytoplasm and nucleus according to the results of the IF analysis (Fig. 5B). These results indicated that HINT1 may be associated with cisplatin sensitivity in SCLC.

To investigate the effect of HINT1 on cisplatin sensitivity in SCLC, we overexpressed HINT1 in cisplatin-resistant SCLC cells and knocked down HINT1 in cisplatin-sensitive SCLC cells (Fig. S4A–D). We then examined the sensitivity of these cells to cisplatin using CCK-8 assays. The results showed that HINT1 overexpression significantly decreased the cisplatin IC50 value in cisplatin-resistant cells, while HINT1 knockdown significantly increased the cisplatin IC50 value in cisplatin-sensitive cells (Fig. 5C). We further analyzed the effect of HINT1 on the level of cisplatin-induced apoptosis. Flow cytometry revealed that the proportion of apoptotic cells was significantly increased in the HINT1-overexpressing group than in the control group under cisplatin treatment (Figs. 5D, E and S5A, B). Western blotting revealed that the expression of the proapoptotic protein BAX was increased and that the expression of the antiapoptotic protein BCL-2 was decreased in cells overexpressing HINT1 (Figs. 5F and S5C). In contrast, the proportion of cisplatin-induced apoptotic cells was significantly lower in the HINT1-silenced cells than in the control cells, and the results of the Western blot analysis of apoptosis-related proteins were consistent with the flow cytometry results (Figs. 5D–F and S5A–C).

**Fig. 6:**
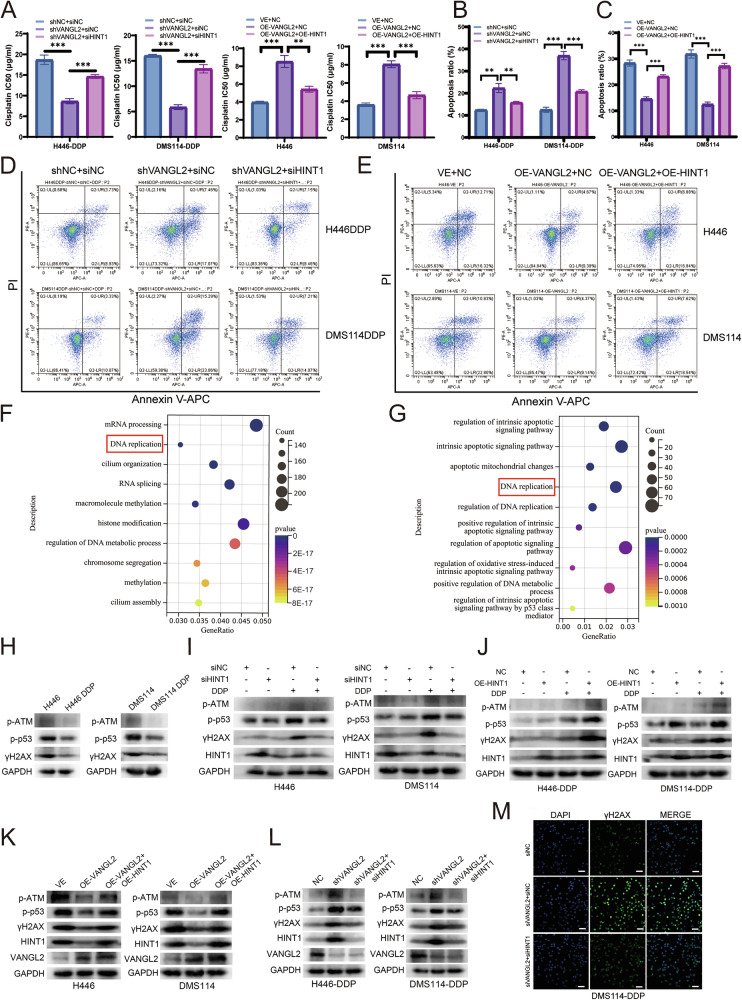
VANGL2 reduced cisplatin-induced ATM and P53 phosphorylation by regulating HINT1. **A** CCK-8 assays showed the effect of simultaneous knockdown or overexpression of VANGL2 and HINT1 on cisplatin IC50 for SCLC cells. ^**^P < 0.01, ^***^P < 0.001. **B**–**E** Flow cytometry assays showed the cisplatin-induced apoptosis after simultaneous knockdown or overexpression of VANGL2 and HINT1 in SCLC cells. ^**^P < 0.01, ^***^P < 0.001. **F** GO functional enrichment analysis of genes positively associated with VANGL2. **G** GO functional enrichment analysis of HINT1 negatively related genes. **H** The protein levels of p-ATM, p-p53, and γH2AX in cisplatin-sensitive and cisplatin-resistant SCLC cells were measured after cisplatin treatment. **I** Cisplatin-sensitive SCLC cells were transfected with siHINT1 or siNC. Cell lysates were collected from cells which untreated or treated with cisplatin. Western blot assays detected the protein levels of p-ATM, p-p53, and γH2AX. **J** Cisplatin-resistant SCLC cells were transfected with HINT1 overexpression plasmid (OE-HINT1) or NC. Cell lysates were collected from cells which untreated or treated with cisplatin. Western blot assays detected the protein levels of p-ATM, p-p53, and γH2AX. **K** There were three groups of transfected cells: NC group (VE), VANGL2 overexpression group (OE-VANGL2), and both VANGL2 and HINT1 overexpression group (OE-VANGL2 and OE-HINT1). Western blot assays detected the expression of p-ATM, p-p53, and γH2AX in three groups of cells treated with cisplatin for 24 h. **L** There were three groups of transfected cells: NC group, VANGL2 knockdown group (shVANGL2), and both VANGL2 and HINT1 knockdown group (shVANGL2 and siHINT1). Western blot assays detected the expression of p-ATM, p-p53, and γH2AX in three groups of cells treated with cisplatin for 24 h. **M** IF assays showed γH2AX expression in SCLC cisplatin-resistant cells treated with cisplatin for 24 h, following silencing with siNC, si-VANGL2, or both si-VANGL2 and si-HINT1. Scale bar = 50 µm.

Further rescue experiments detected that when VANGL2 expression was downregulated, the sensitivity of SCLC cells to cisplatin increased, and the cisplatin IC50 decreased. However, when both VANGL2 and HINT1 were downregulated simultaneously, the increased cisplatin sensitivity caused by VANGL2 downregulation was restored (Fig. 6A). In addition, cisplatin resistance in SCLC cells increased when VANGL2 was overexpressed, whereas the increased cisplatin resistance caused by VANGL2 overexpression was restored when both VANGL2 and HINT1 were overexpressed simultaneously (Fig. 6A).

Next, we performed flow cytometry assays to analyze the effect of HINT1 rescue on cisplatin-induced apoptosis. When VANGL2 was knocked down, the rate of cisplatin-induced apoptosis increased, whereas when both VANGL2 and HINT1 were knocked down, the increased rate of cisplatin-induced apoptosis caused by VANGL2 knockdown was restored (Fig. 6B, D). In addition, the decrease in cisplatin-induced apoptosis in cells with stable VANGL2 overexpression was rescued by transfecting plasmids that overexpress HINT1 (Fig. 6C, E).

Together, these results indicated that VANGL2 affected cisplatin-induced apoptosis and promoted cisplatin resistance in SCLC by downregulating HINT1.

### HINT1 regulates cisplatin-induced activation of the ATM-p53 pathway

To further investigate the pathways by which VANGL2 and HINT1 affect cisplatin resistance in SCLC, we downloaded the IMPOWER133 cohort, which contains transcriptomic sequencing data from 271 SCLC patients. We performed a difference analysis between the VANGL2-high group and the VANGL2-low group according to the median expression. The upregulated genes were subsequently subjected to GO enrichment analysis, and the results revealed that the upregulated genes were enriched in the DNA damage repair pathway (Fig. 6F), suggesting that the high expression of VANGL2 may be associated with DNA damage repair. We further investigated the potential function of HINT1 using the IMPOWER133 cohort. GO enrichment analysis of genes negatively related to HINT1 revealed that the downregulated genes could also be enriched in the DNA damage repair pathway (Fig. 6G), suggesting that low HINT1 expression may be associated with DNA damage repair.

Previous studies have suggested that ATM is an upstream DNA damage response (DDR) kinase that is rapidly phosphorylated at Ser1981 and activated upon DNA damage [15]. When severe genotoxic stress occurs, ATM induces p53 phosphorylation at Ser46, which activates the p53 apoptosis pathway and leads to apoptosis [16]. In addition, activated ATM can recruit to DNA damage sites and rapidly phosphorylate the DNA damage repair-associated protein H2AX, resulting in the production of γH2AX [17], which can be used to measure the extent of DNA damage [18]. Therefore, we analyzed the phosphorylation levels of ATM, p53, and H2AX proteins in cisplatin-sensitive and cisplatin-resistant SCLC cells after cisplatin treatment. Western blotting revealed significantly higher expression levels of p-ATM (Ser1981), p-p53 (Ser46), and γH2AX in cisplatin-sensitive cells than in cisplatin-resistant cells, indicating that the degree of DNA damage induced by cisplatin was more severe in cisplatin-sensitive cells (Fig. 6H). We further extracted cellular proteins from HINT1-knockdown or HINT1-overexpressing cells treated with cisplatin or the PBS control for 24 h. Western blotting revealed decreased expression of p-ATM (Ser1981), p-p53 (Ser46) and γH2AX in HINT1-knockdown cells under cisplatin stimulation (Fig. 6I), whereas the expression of p-ATM (Ser1981), p-p53 (Ser46) and γH2AX in HINT1-overexpressing cells increased after cisplatin treatment (Fig. 6J).

Together, these results suggested that HINT1 may be a promoter of cisplatin-induced activation of the ATM-p53 pathway.

### VANGL2 regulation of HINT1 levels contributes to SCLC cisplatin resistance by regulating the ATM-p53 pathway

To further clarify whether VANGL2 is involved in the regulation of the ATM-p53 pathway by modulating HINT1, we performed rescue experiments by extracting total protein from cells treated with cisplatin for 24 h. When VANGL2 and HINT1 were simultaneously overexpressed in chemosensitive cells, the decreases in p-ATM (Ser1981), p-p53 (Ser46), and γH2AX protein expression caused by VANGL2 overexpression were reversed (Fig. 6K). In contrast, the increased expression of p-ATM (Ser1981), p-p53 (Ser46) and γH2AX caused by VANGL2 knockdown was restored when both VANGL2 and HINT1 were knocked down simultaneously in chemoresistant cells (Fig. 6L).

In addition, we transfected cisplatin-resistant SCLC cells with control siNC, si-VANGL2, and co-transfection of si-VANGL2 and si-HINT1, respectively. Then, we added cisplatin for 24 h after transfection and performed IF experiments to observe the fluorescence intensity of γH2AX, an indicator of DNA damage. IF experiments revealed that simultaneous knockdown of VANGL2 and HINT1 reversed the increase in γH2AX fluorescence intensity caused by VANGL2 downregulation (Figs. 6M and S6A, B).

These experimental data suggested that VANGL2 regulation of HINT1 levels made a contribution to SCLC cisplatin resistance by regulating the ATM-p53 pathway.

## Discussion

SCLC is an aggressive subtype of lung cancer with a poor prognosis. Cisplatin is a first-line drug for the treatment of SCLC. However, although most patients with SCLC have a favorable response to initial cisplatin therapy, the majority of patients develop cisplatin resistance within a short period of time. Unfortunately, there is no effective second-line therapeutic strategy, resulting in a poor clinical prognosis for SCLC patients once cisplatin resistance develops. Therefore, elucidating the mechanism underlying cisplatin resistance in SCLC is critical for improving the clinical management of SCLC patients.

VANGL2, which was first reported in Drosophila studies, is highly conserved among different species [19]. In mammals, VANGL2 regulates planar polarity and development in multiple organs, including brain, lung, and kidney [20, 21]. VANGL2 has been found to be closely associated with female reproductive system tumors and is elevated in breast, ovarian, and endometrial cancers [7]. Previous studies have reported that VANGL2 promotes breast cancer invasion and metastasis [8]. In addition, VANGL2 can bind to p62/SQSTM1 to form a complex that activates the JNK signaling pathway, thereby promoting the proliferation of breast cancer cells [9]. However, the relationship between VANGL2 and cisplatin resistance has not been investigated. In this study, we demonstrated for the first time that VANGL2 could promote cisplatin resistance in SCLC. We selected the VANGL2 gene by transcriptome sequencing analysis and discovered that VANGL2 was highly expressed in cisplatin-resistant SCLC cells through in vitro and in vivo experiments. In addition, differential VANGL2 expression affected the IC50 and apoptosis of SCLC cells treated with cisplatin.

To explore the possible downstream mechanisms by which VANGL2 promotes cisplatin resistance in SCLC, we applied immunoprecipitation combined with tandem mass spectrometry to identify interacting proteins. On the basis of a comprehensive analysis of previous literature and in vitro experiments, we discovered that HINT1 was regulated by VANGL2 and that this regulation occurred mainly at the post-translational level of the protein. The ubiquitin-proteasome and autophagy-lysosome pathways are the two major protein degradation pathways in eukaryotic cells [22]. Previous studies have shown that VANGL2 directly binds to LAMP-2A and promotes LAMP-2A degradation to regulate molecular chaperone-mediated autophagy in MSCs [23]. Furthermore, Hu et al. [24] reported that VANGL2 promotes selective autophagy of TBK1 via the E3 ligase TRIP. We further investigated the pathway by which VANGL2 regulated HINT1 and found that the autophagy inhibitor chloroquine rescued the decreased expression of HINT1 caused by VANGL2 overexpression. These results suggested that VANGL2 may degrade HINT1 via the autophagy-lysosome pathway, which is consistent with previous reports of the autophagy-regulatory function of VANGL2 [23, 24].

Histidine triad nucleotide binding protein 1 (HINT1) is a tumor suppressor that inhibits the proliferation of several tumors, including malignant melanoma [25–27], hepatocellular carcinoma [28–30], non-SCLC [31], and colon cancer [26, 32, 33]. It has been reported that HINT1-deficient mice have an increased resistance to ionizing radiation [34]. High expression of HINT1 in human breast and colon cancer leads to caspase-3 activation, cytochrome C release, and DNA breakage. In addition, HINT1 is able to upregulate the proapoptotic proteins P53 and BAX and downregulate the expression of the antiapoptotic protein BCL-2 [35]. However, there are no previous reports on whether HINT1 is involved in tumor resistance to cisplatin. In this study, we found that HINT1 negatively regulated cisplatin resistance in SCLC. This finding is critical for the future development of drugs that can reverse SCLC cisplatin resistance.

To further characterize the mechanisms by which VANGL2 and HINT1 regulate cisplatin resistance in SCLC, we performed GO enrichment analysis and found that the DDR pathway was highly enriched. Previous studies have shown that cisplatin is an inducer of DNA damage and that its toxicity to tumors is mediated by the formation of intra- and interstrand DNA crosslinks, as well as DNA-protein crosslinks [36]. The interaction of cisplatin with DNA inhibits the progression of replicative DNA polymerases, leading to the formation of a highly toxic form of DNA damage, namely, double-strand breaks (DSBs) [37]. ATM is an upstream DDR kinase that acts as a DNA damage sensor. When cisplatin-induced DNA damage occurs, ATM is rapidly autophosphorylated at serine 1981, resulting in the activation of ATM kinase activity [12]. Activated ATM can directly phosphorylate p53 at Ser15 and Ser46, and it induces Ser46 phosphorylation when severe genotoxic stress occurs [13]. The p53 Ser46 phosphorylation is strongly associated with irreparable DNA damage and is able to control a distinct set of proapoptotic target genes, including BAX, p53AIP1, p53INP1, NOXA, and PTEN, and ultimately leads to the apoptosis of tumor cells [16]. In addition, ATM can rapidly phosphorylate the histone variant H2AX at DSBs and produce γH2AX, which is often used as a measure of the extent of DNA damage [18].

**Fig. 7:**
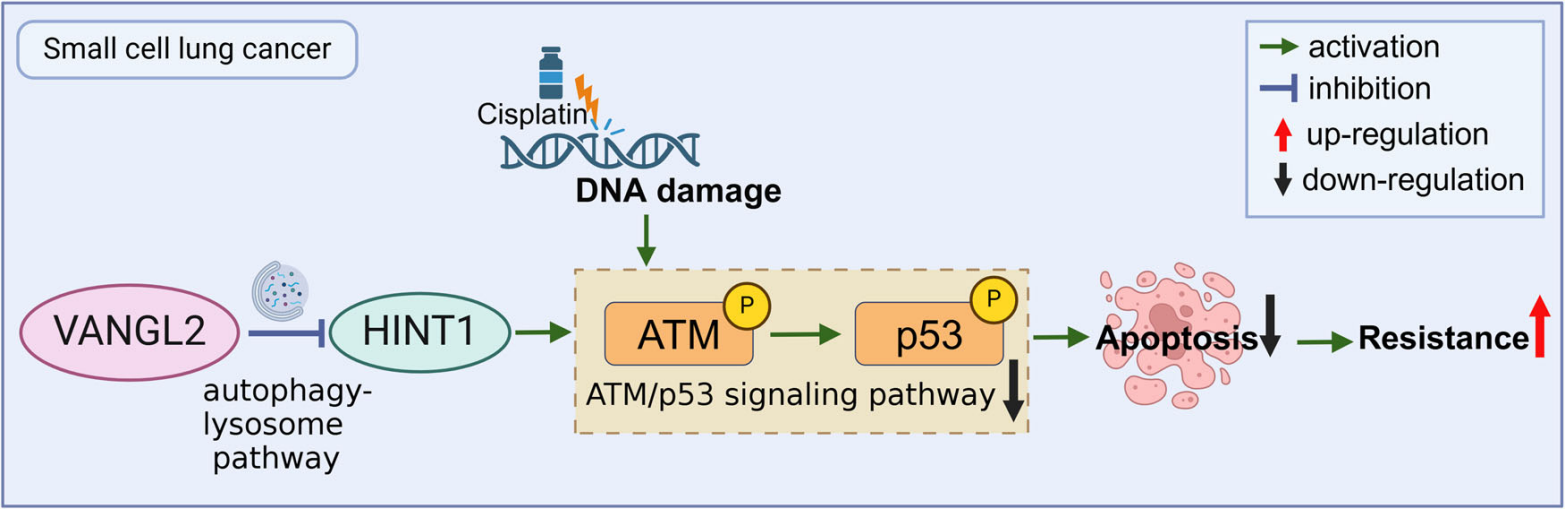
Mechanism diagram: VANGL2 regulates the ATM-p53 pathway-mediated apoptotic response of SCLC to cisplatin through downregulation of HINT1. This figure is created by biorender.

Previous reports have shown that HINT1-deficient cells are defective in the DDR pathway, with reduced phosphorylation of ATM, p53, and H2AX. HINT1 binds to ATM, and this association is increased after DNA damage. HINT1 is important for mediating and coordinating the induction of early (ATM activation) and late (γH2AX removal) steps in the DDR [14]. Our findings revealed that cisplatin-induced activation of the ATM-p53 pathway was more pronounced in cisplatin-sensitive cells than in cisplatin-resistant cells and that the phosphorylation of ATM and p53 was regulated by VANGL2 and HINT1. Mechanistically, in cisplatin-resistant cells, VANGL2 overexpression leads to the autophagic degradation of HINT1, and decreased HINT1 expression further reduces the phosphorylation response of ATM and p53 to cisplatin-induced DNA damage. The decreased phosphorylation of p53 leads to the inhibition of downstream apoptotic pathways, resulting in a reduction in cisplatin-induced apoptosis. These events ultimately lead to cisplatin resistance in SCLC (Fig. 7). These findings suggest that VANGL2 may be a promising clinical target for reversing cisplatin resistance in SCLC. Our study still has limitations, relying mainly on SCLC cell lines for in vitro and in vivo experimental validation of the role of VANGL2 in SCLC cisplatin resistance. Although primary SCLC cells can provide a more representative model, their procurement and transfection are challenging. Future studies will need to collect more tumor tissue samples from SCLC patients to construct PDX models to better verify the function of VANGL2 in SCLC.

## Materials and methods

### Cell lines

The human-derived SCLC cell lines DMS114, H446, H146, and H69 were purchased from ATCC (American Type Culture Collection, USA). The multidrug-resistant H69AR cell line was also obtained from ATCC. The cisplatin-resistant SCLC cell lines DMS114-DDP, H446-DDP, and H146-DDP were developed in our laboratory by incubating SCLC cells with increasing doses of cisplatin for at least 6 months. Cisplatin-resistant cell lines were cultured with cisplatin to maintain their cisplatin resistance.

### Cell transfection

The cells were stably transduced with lentiviral vectors containing shVANGL2 and the VANGL2 sequence (Shanghai Genechem Co., Ltd.). To achieve stable expression, cells were first seeded in six-well plates. After the medium was replaced with fresh medium, infection-enhancing medium, and the appropriate lentivirus were added. The six-well plate was incubated for 16 h, and then the medium was replaced with fresh complete medium. Cells transfected with lentivirus were expanded and selected with puromycin. The expression of target genes in cells with stable lentiviral transfections after puromycin selection was confirmed via qRT-PCR and Western blotting. The cells were transiently transfected with HINT1 siRNA (Guangzhou Hanyi Biotechnology Co., Ltd.) using PepMute™siRNA Transfection Reagent (SignaGen, USA) according to the manufacturer’s instructions. The sequences of the shRNAs and siRNAs used in this study are listed in Tables S1 and S2.

### Cell viability assay

The cellular response to cisplatin was evaluated using CCK-8 reagent (CCK-8, Dojindo, Kumamoto, Japan). Cells were inoculated into 96-well plates at 8000 cells per well (DMS114 and DMS114-DDP) or 15000 cells per well (H446 and H446-DDP). The cells were then incubated with various concentrations of cisplatin. After 24 h of incubation, the medium was replaced with RPMI 1640 medium containing 10% CCK-8 solution, and the plates were incubated for 1–4 h. The absorbance of each well was measured at 450 nm using a microplate reader. The half-maximal inhibitory concentration (IC50) of cisplatin was calculated from the OD value.

### Flow cytometry

Transfected cells were treated with cisplatin for 24 h to induce apoptosis, and the cells were collected for apoptosis experiments. The dose of cisplatin was equivalent to 1/2–1/3 of the IC50 of SCLC cells. Apoptosis was evaluated using Annexin V-PI Apoptosis Detection Reagent (Elabscience, Wuhan, China).

### Quantitative real-time reverse transcription PCR (qRT-PCR)

Cells were collected, and RNA was extracted sequentially with TRIzol, chloroform, and isopropanol. The samples were washed with 80% ethanol, and DEPC was added to dissolve the RNA precipitate after complete evaporation of the ethanol. The concentration of RNA was determined using a UV spectrophotometer. Reverse transcription was performed using Reverse Transcription Master Mix (EZBioscience, A0010). The RNA levels were quantified via SYBR Green qPCR Master Mix (EZBioscience, A0001-R1). Primer information is provided in Table S3.

### Immunoblot analysis

Total proteins were extracted using RIPA buffer (Hangzhou Fode Biotechnology Co., Ltd.) containing a mixture of protease and phosphatase inhibitors. Protein concentrations were measured via the BCA assay. Proteins of different molecular weights were run on SDS-PAGE gels and then transferred to PVDF membranes. The membranes were blocked with 5% BSA or 5% skim milk sealant for 2 h after transfer. After blocking, the membranes were incubated with primary antibody overnight at 4 °C and then incubated with secondary antibody for 1.5 h at room temperature. After being washed with TBST, the membranes were coated with enhanced chemiluminescence solution. Images were captured via the ChemiDoc™ Imaging System. Antibody information is provided in Table S4.

### IF staining

The cells were seeded in confocal dishes, and the final cell density in the confocal dish was approximately 50–60%. Cells were fixed with paraformaldehyde, permeabilized with 0.3% Triton X-100, and blocked with 10% goat serum for at least 1 h at room temperature. Then, cells were incubated with primary antibodies diluted in goat serum for at least 12 h. After being washed with PBST, cells were incubated with appropriate fluorescent secondary antibodies for 1 h at room temperature in the dark. DAPI (nuclear staining solution) was added to the samples, which were incubated in the dark for 3 min, and then the samples were washed with PBST. Images were captured with a fluorescence microscope.

### co-IP

Total proteins were collected, and target proteins interacting with the decoy proteins were extracted according to the Pierce Crosslinked Magnetic Bead Immunoprecipitation/Co-IP Kit instructions (Thermo Scientific, 88805). The interacting proteins were analyzed by mass spectrometry (MS) and then verified by Western blotting.

### In vivo study

Four-week-old female BALB/c nude mice were raised in a specific pathogen-free (SPF) environment. SCLC suspensions were prepared, and approximately 1 × 10^7^ cells were injected subcutaneously into the mice according to the experimental design. When tumors reached approximately 5 mm in diameter, mice were randomly assigned to treatment or control groups for in vivo cisplatin sensitivity experiments. The mice were injected with 3 mg/kg cisplatin or an equivalent volume of saline every 4 days. The length and diameter of the subcutaneous tumors were measured with calipers, and the volume was calculated via the following equation: *V* = (length × width × width/2).

### SCLC cohorts

Three public clinical SCLC datasets were included in this study: the GSE149507, GSE60052, and IMPOWER133 cohorts. The GSE149507 dataset contains RNA sequencing data from 18 cases of SCLC tissues and paired adjacent normal lung tissues [38]. The GSE60052 dataset contains whole-genome sequencing data and clinical information from 79 patients with SCLC [39]. After matching the clinical and sequencing data of the SCLC patients, a total of 48 SCLC patients from GSE60052 were available for survival analysis. The IMPOWER133 cohort includes transcriptomic sequencing data from 271 SCLC patients and was used for enrichment analysis [40].

### DEA

RNA-seq data were analyzed using the R software, and DEA was performed via the “limma” package. Gene expression in chemoresistant and chemosensitive cells was analyzed via log2FC values derived from DEA. In this study, log2FC > 0 indicated that the gene was more highly expressed in chemoresistant cells, while log2FC < 0 indicated that the gene was more highly expressed in chemosensitive cells. *P* value < 0.05 indicates a significant association of this gene with chemotherapy resistance.

### Statistical analysis

The R software package “maxstat” was used to define optimal thresholds of the SCLC clinical cohort. Survival analysis and Cox regression analysis were performed using the “survival” and “survminer” packages. In this study, GraphPad Prism 8 software was used to analyze and visualize the results. The results of three independent experiments were expressed as the mean ± standard deviation (S.D. ± mean). Student’s *t*-test and Wilcoxon test were used to determine the significance of differences between two groups of normally distributed data and between two groups of non-normally distributed data, respectively. *P* value < 0.05 was considered statistically significant. ^*^, ^**^, ^***^, and ^****^ indicate *P* < 0.05, *P* < 0.01, *P* < 0.001, and *P* < 0.0001, respectively.

## Supplementary information

Original WB figures

Supplementary Material

## Data Availability

The datasets generated during and/or analyzed during the current study are available from the corresponding author on reasonable request.
