# Supplementary material for: VANGL2 downregulates HINT1 to inhibit the ATM-p53 pathway and promote cisplatin resistance in small cell lung cancer
Source: Cell Death Discov. 2025 Apr 8;11:153. doi: 10.1038/s41420-025-02424-w (PMC11979007; doi:10.1038/s41420-025-02424-w)
Supplement: Supplementary file 1 — Original WB figures [file 41420_2025_2424_MOESM1_ESM.docx]

# fig1G

## H69AR

HSP90


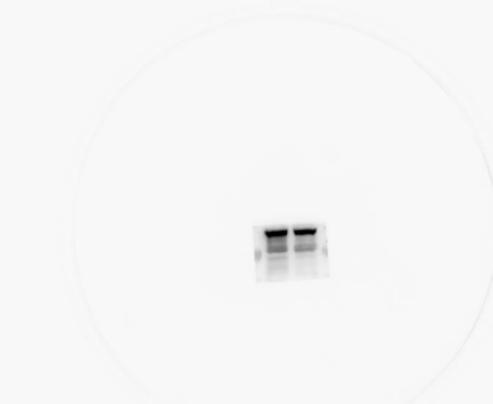


VANGL2


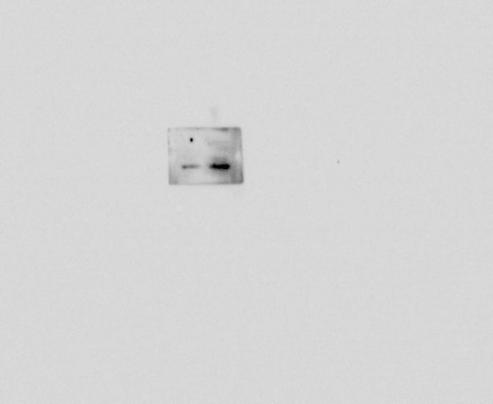


## DMS114DDP

HSP90


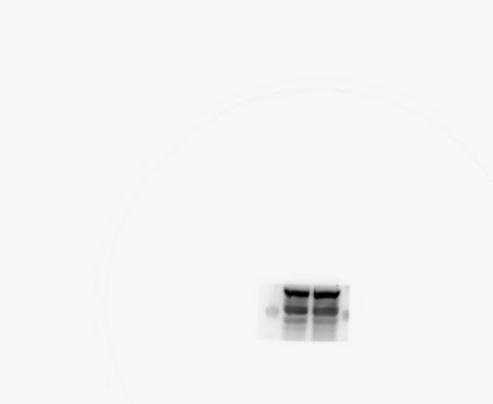


VANGL2


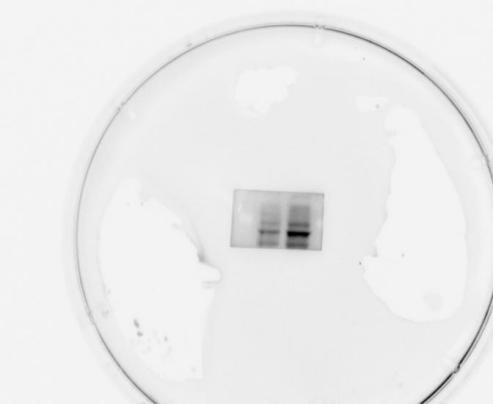


## H146DDP

HSP90


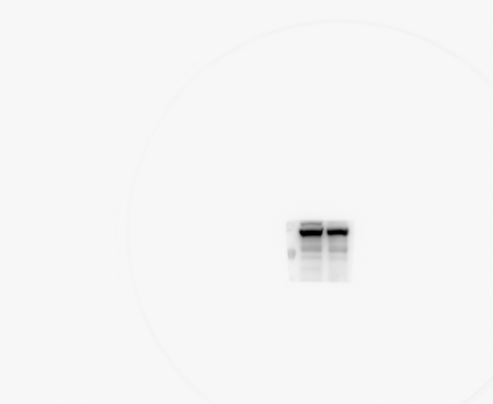


VANGL2


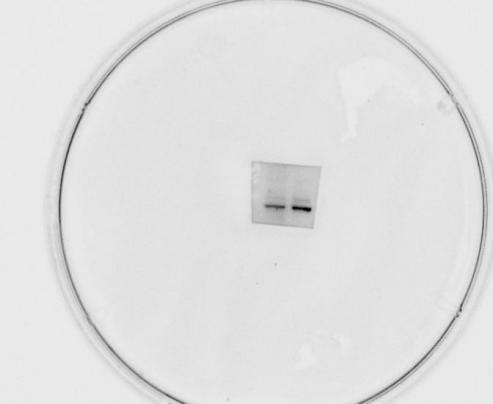


## H446DDP

HSP90


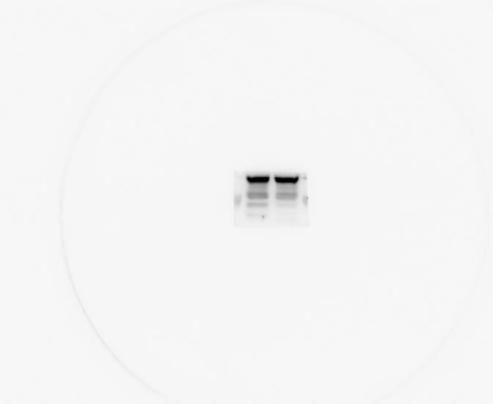


VANGL2


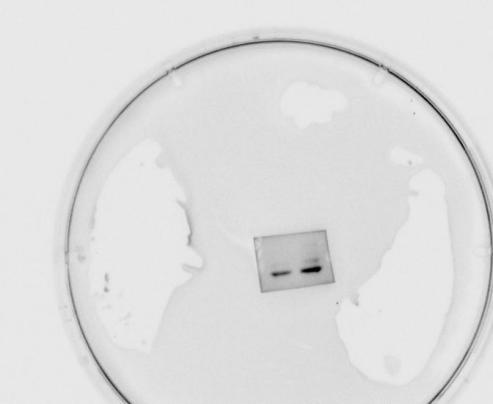


# fig2D


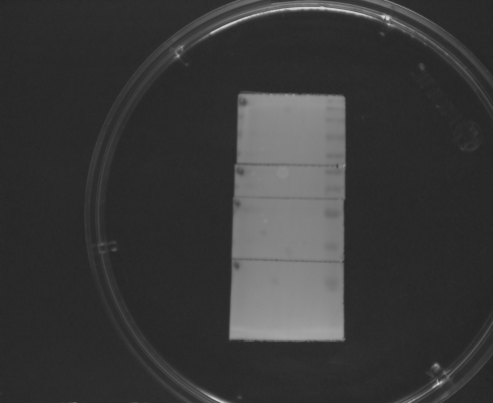


BAX


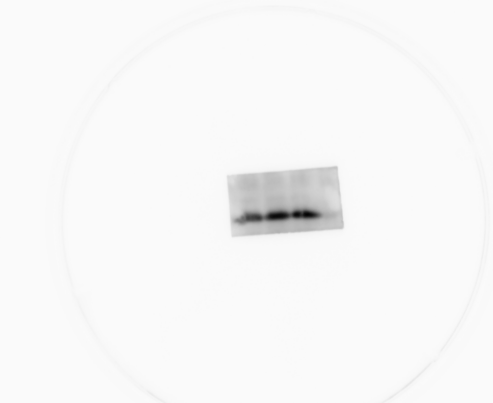


BCL2


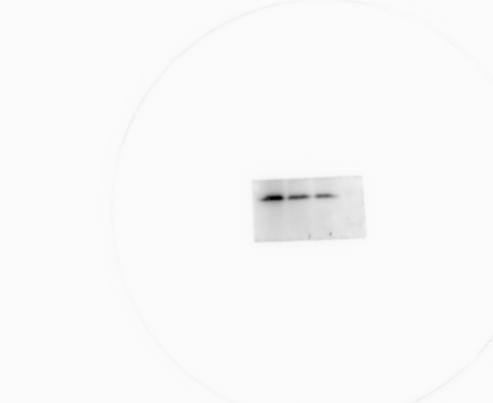


GAPDH


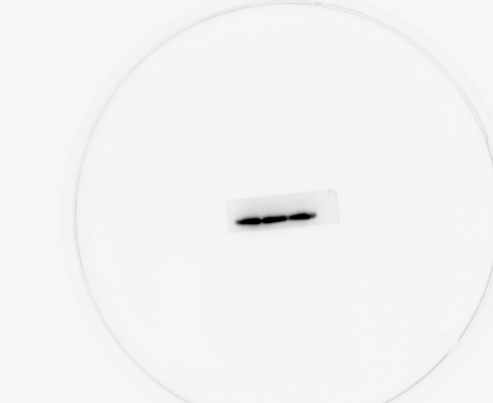


VANGL2


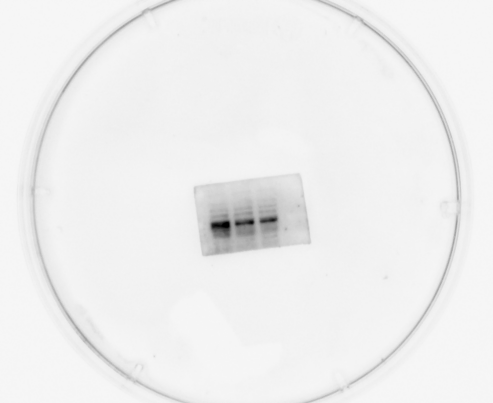


# fig2H


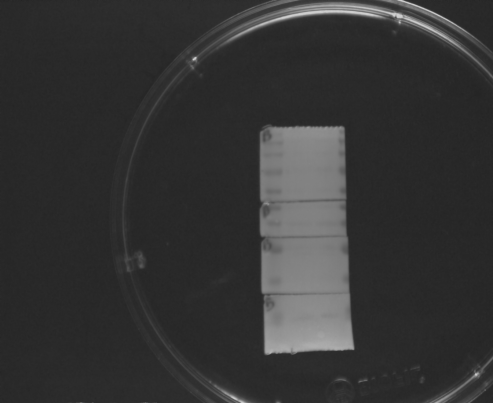


BAX


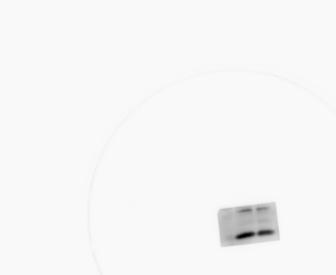


BCL2


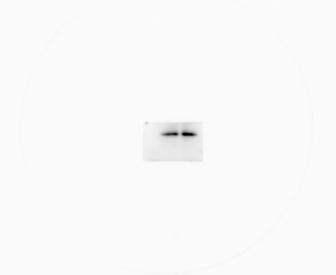


GAPDH


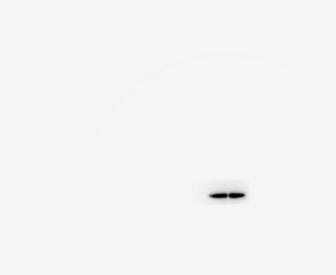


VANGL2


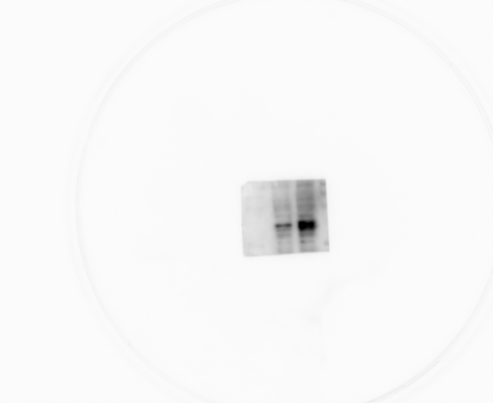


# fig4B

## H446-DDP-IP:VANGL2


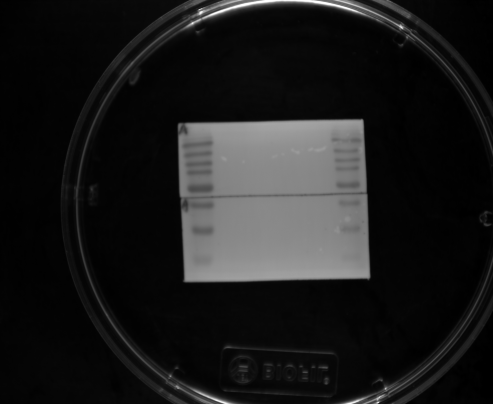


HINT1


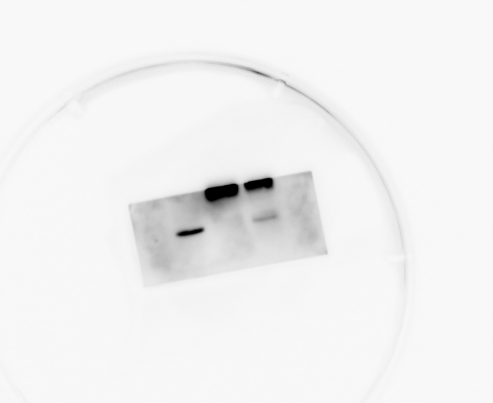


VANGL2


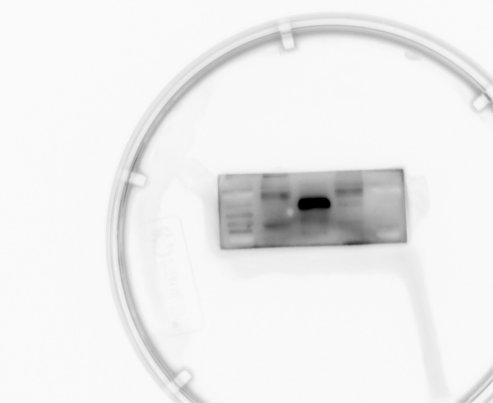


## H446-DDP-IP:HINT1


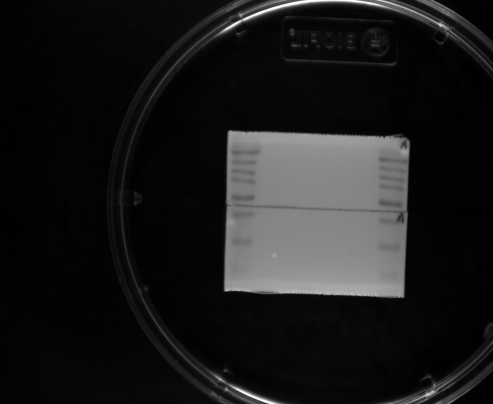


HINT1


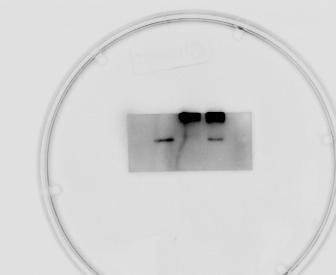


VANGL2


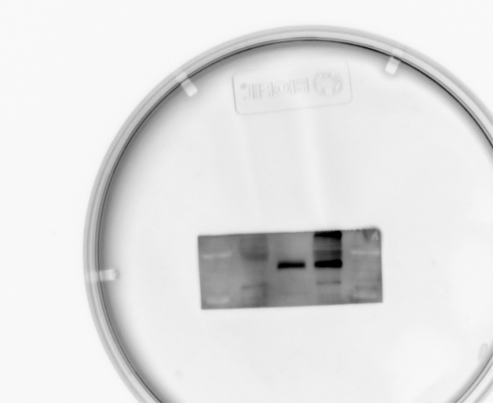


# fig4C

## DM114-DDP-IP:VANGL2


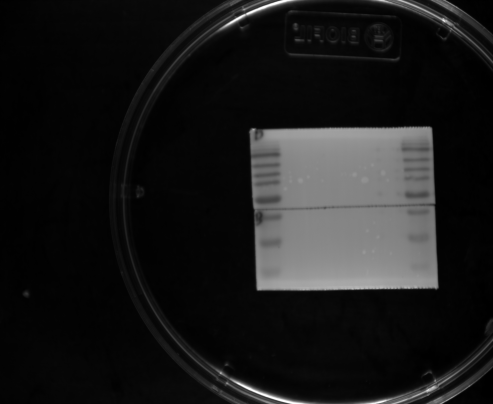


HINT1


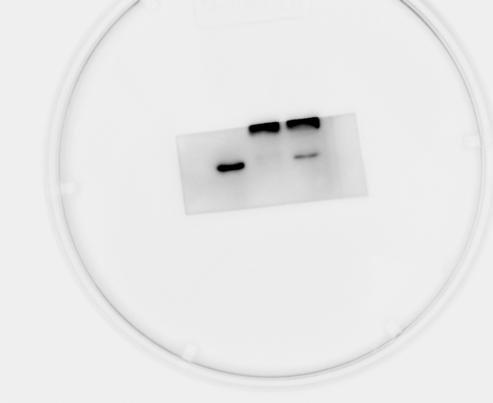


VANGL2


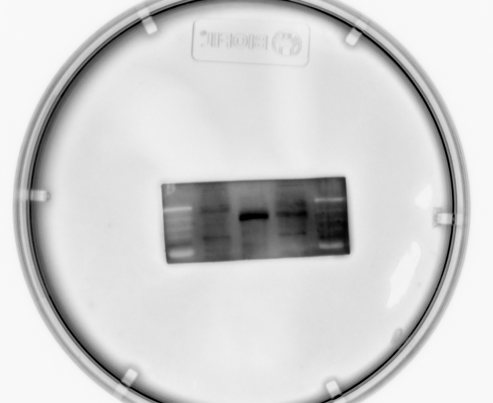


## DM114-DDP-IP:HINT1


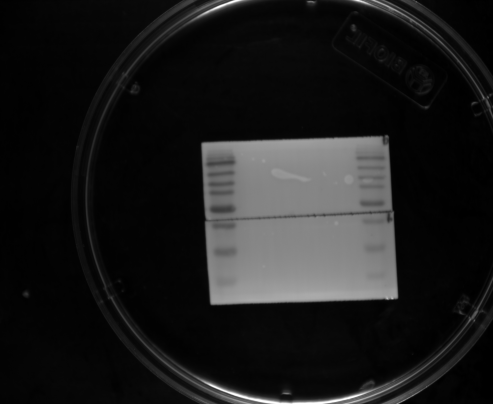


HINT1


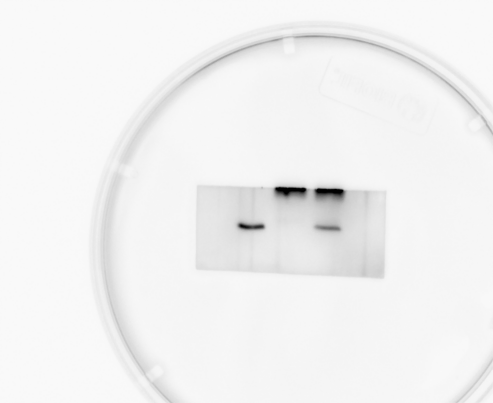


VANGL2


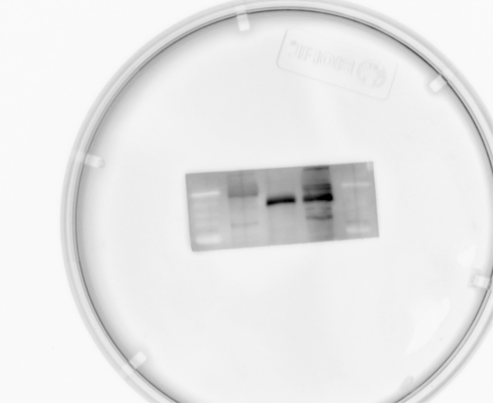


# fig4E

## DMS114-DDP


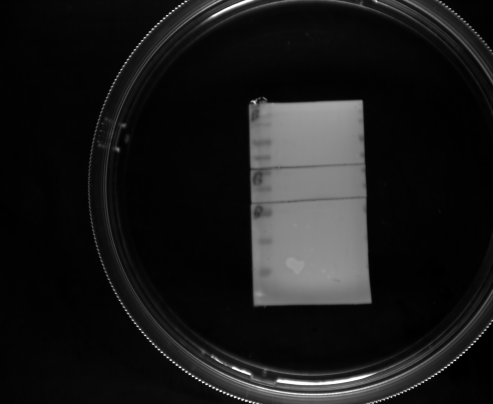


GAPDH


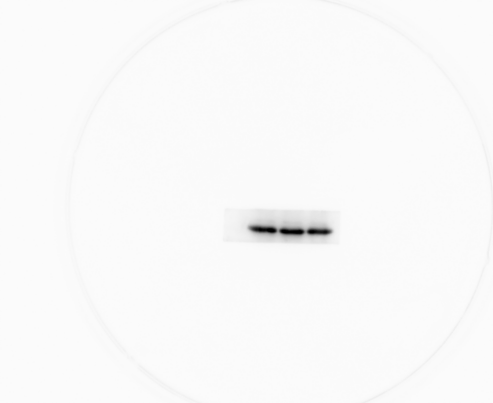


HINT1


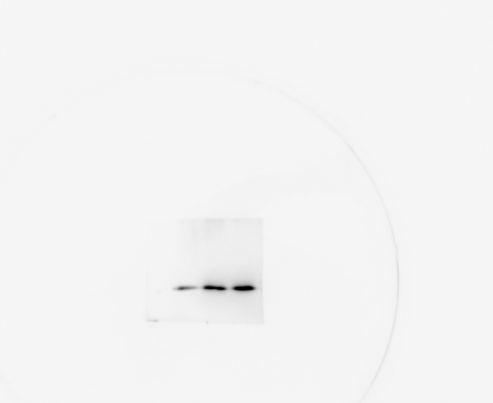


VANGL2


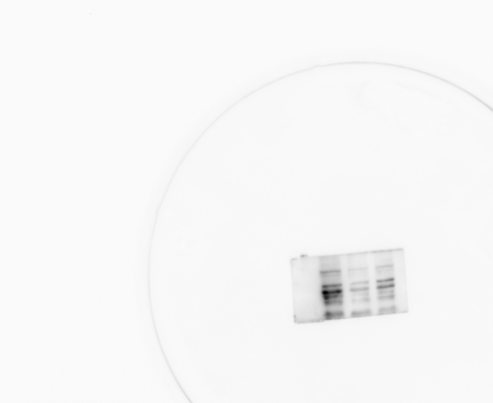


## H446-DDP


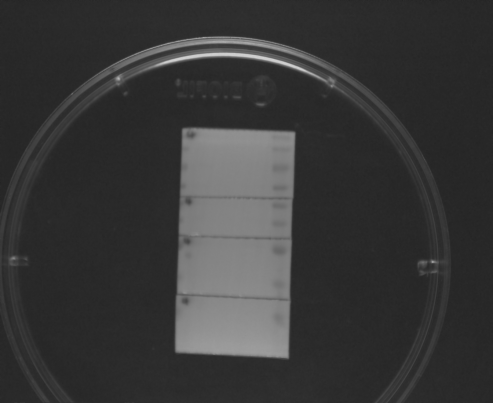


GAPDH


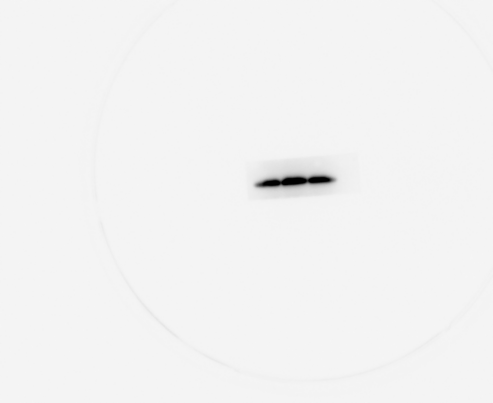


HINT1


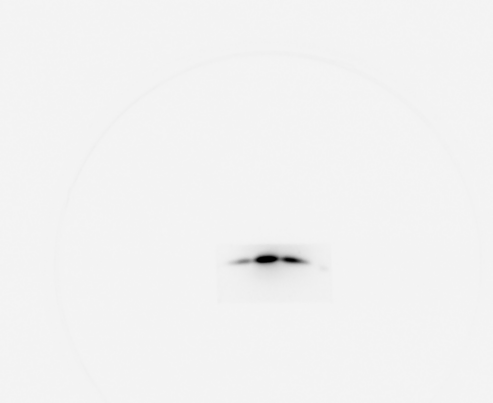


VANGL2


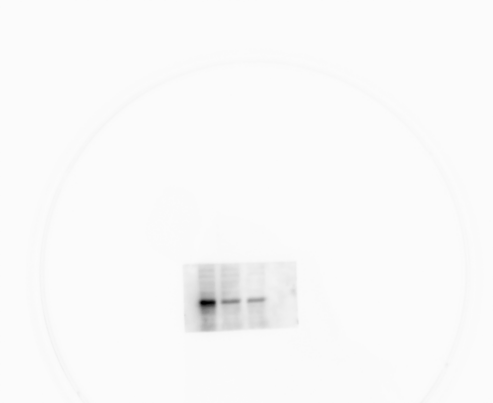


# fig4F

## DMS114


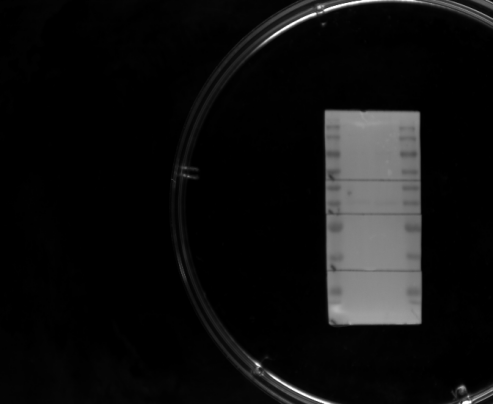


GAPDH


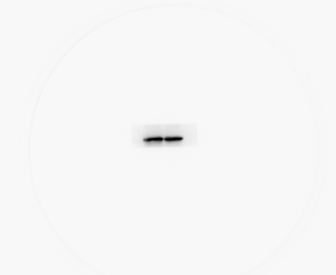


HINT1


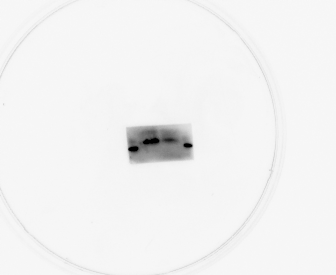


VANGL2


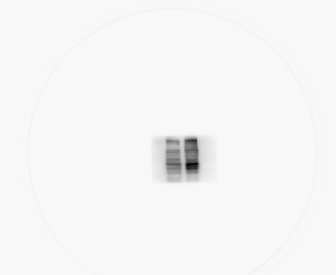


## H446


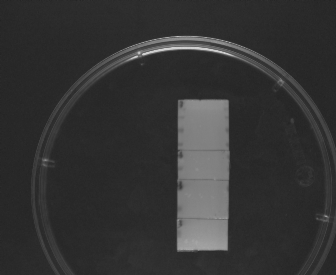


GAPDH


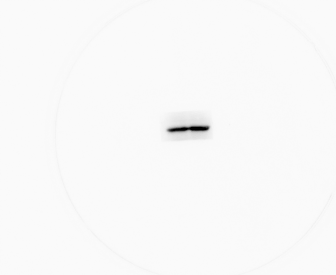


HINT1


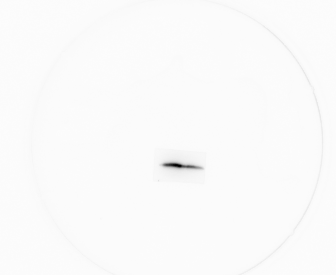


VANGL2


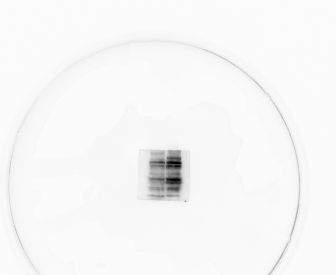


# fig4G

## DMS114


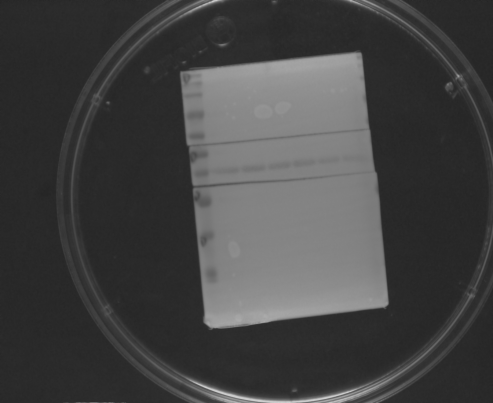


GAPDH


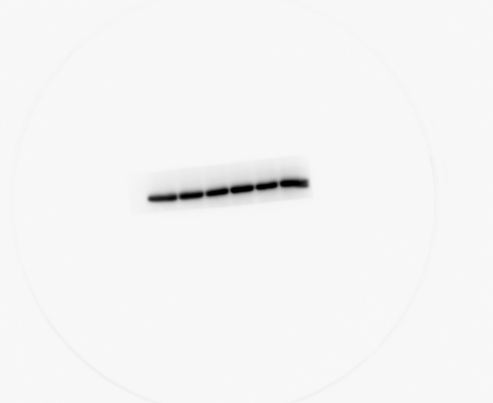


HINT1


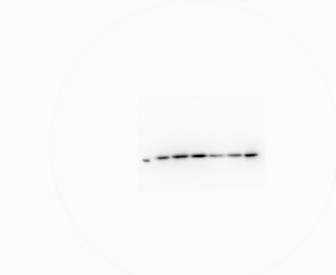


VANGL2


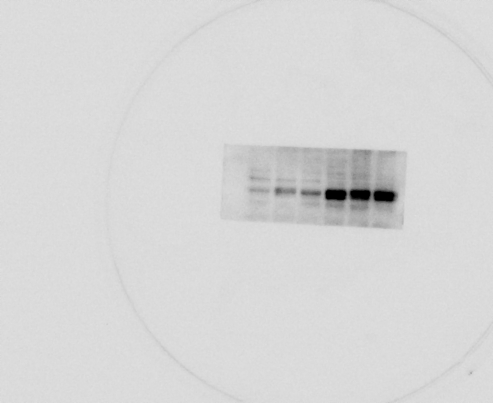


# H446


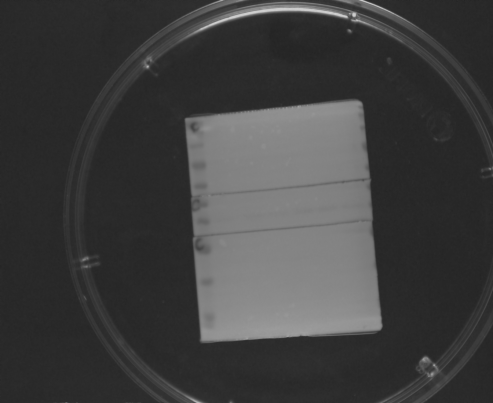


GAPDH


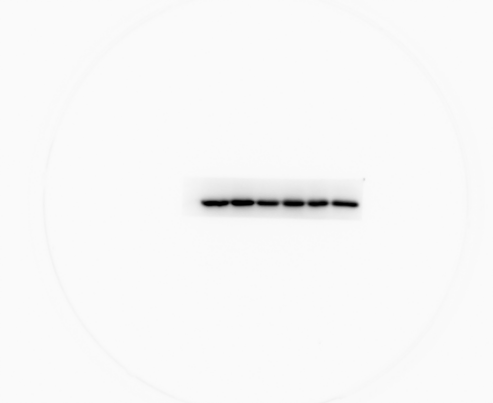


HINT1


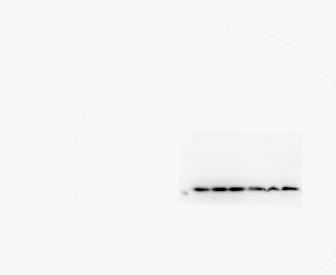


VANGL2


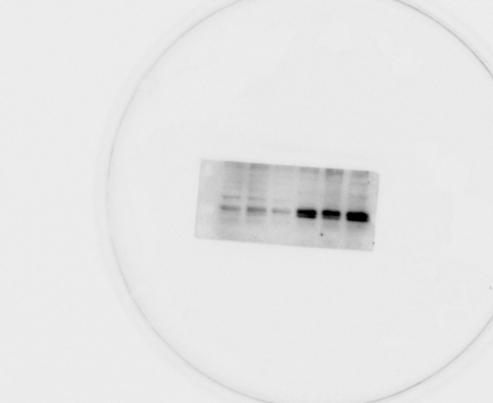


# fig5A

## DMS114


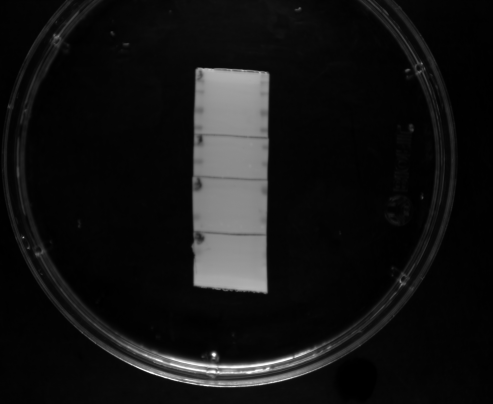


GAPDH


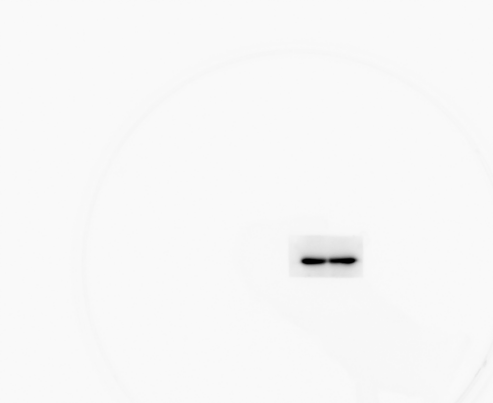


HINT1


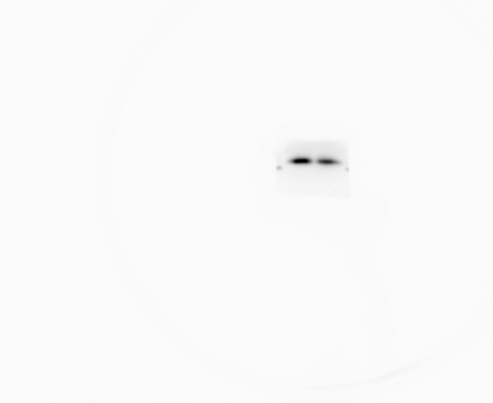


## H446


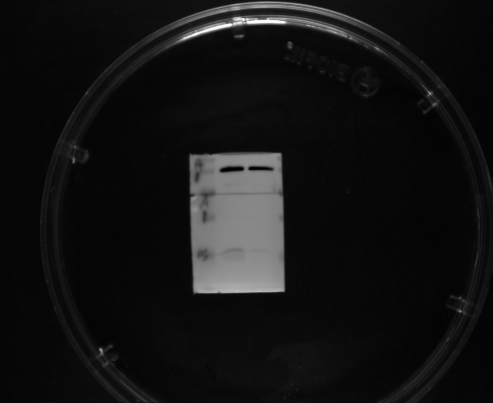


GAPDH


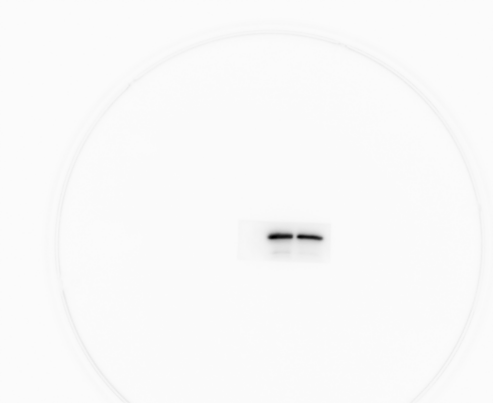


HINT1


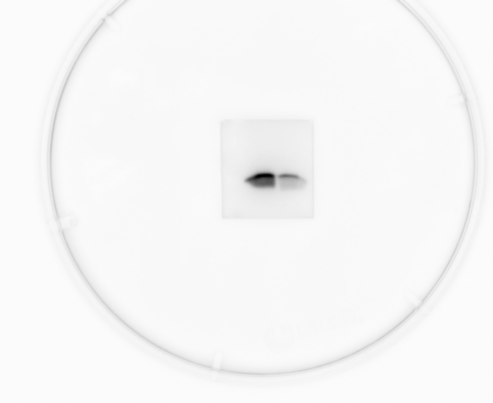


# fig5F

## H446-DDP


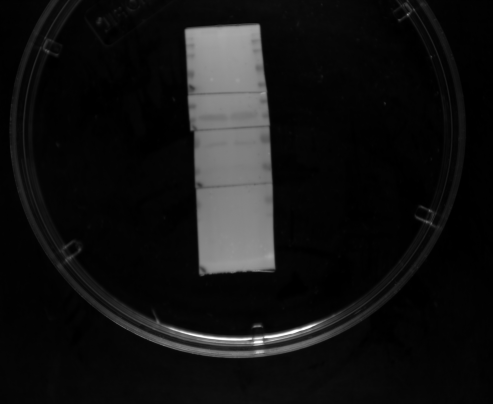


BAX


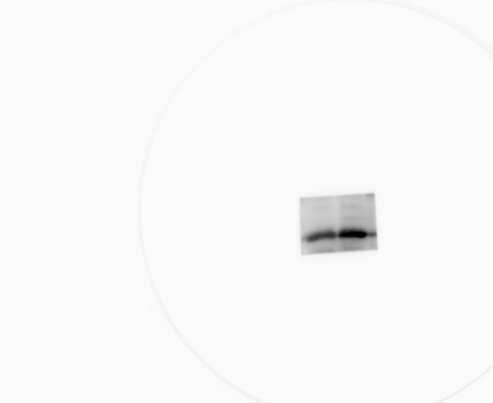


BCL2


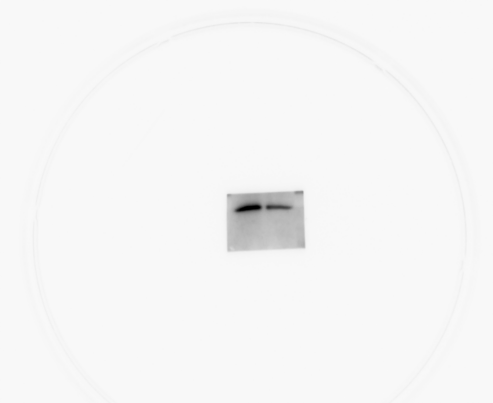


GAPDH


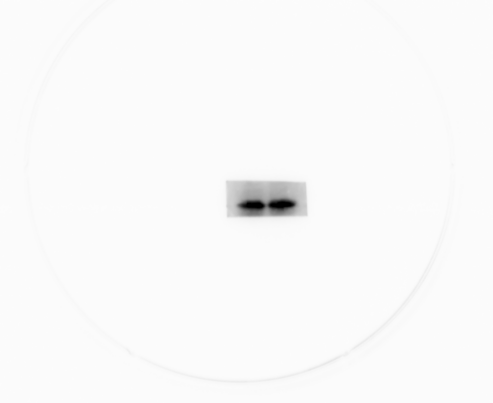


HINT1


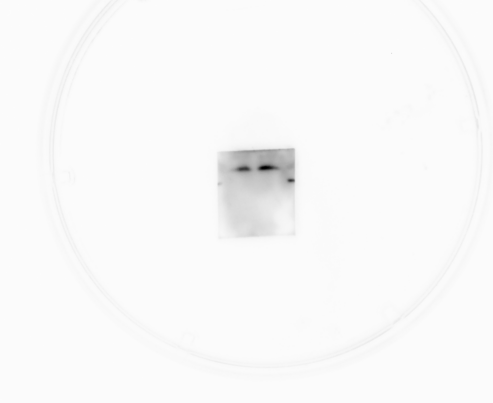


## H446


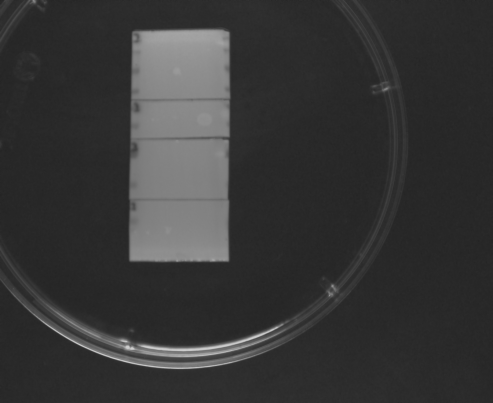


BAX


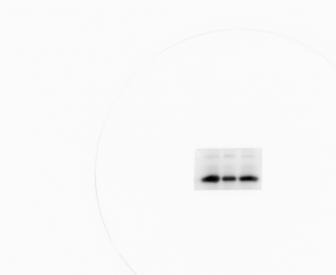


BCL2


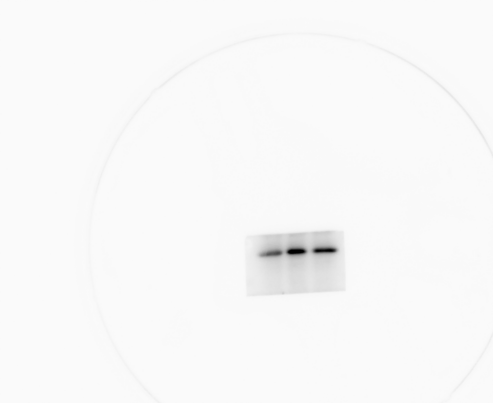


GAPDH


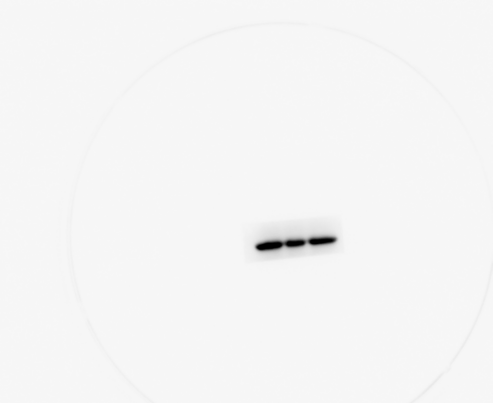


HINT1


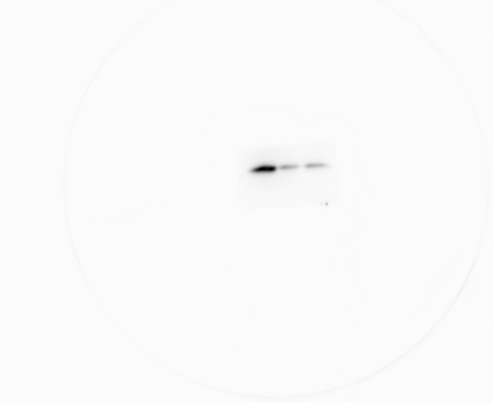


# fig6H

## 114N-DDP


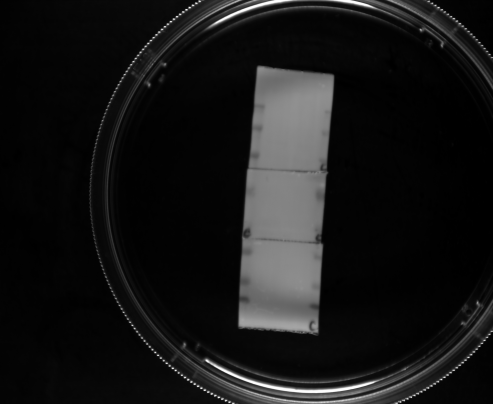


GAPDH


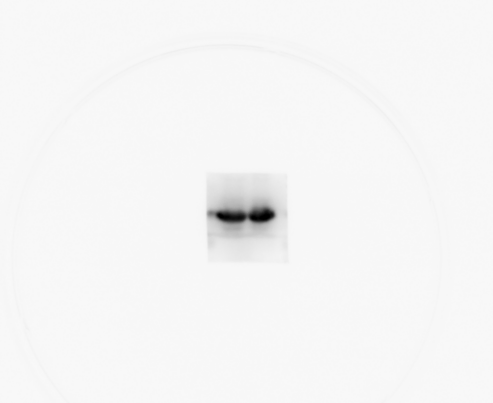


P-ATM


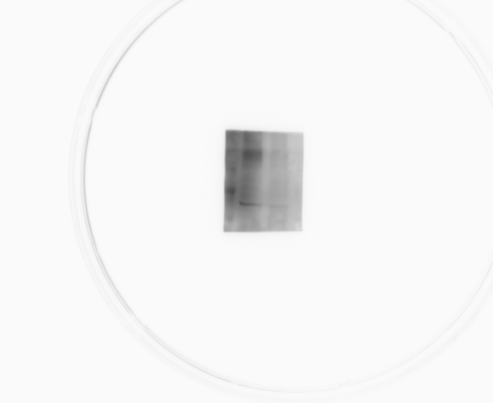


P-P53


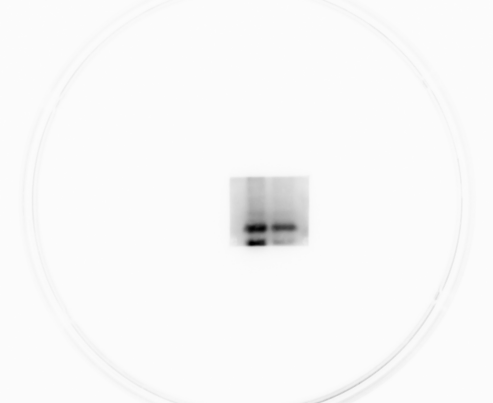


γH2AX


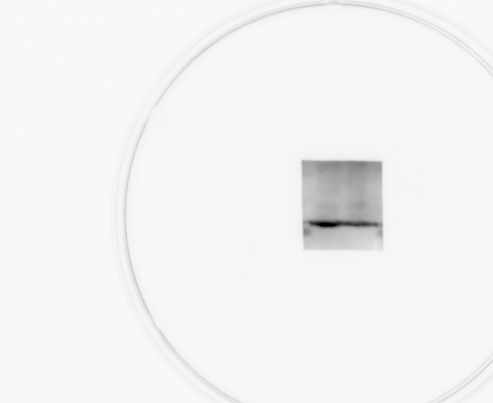


## H446N-DDP


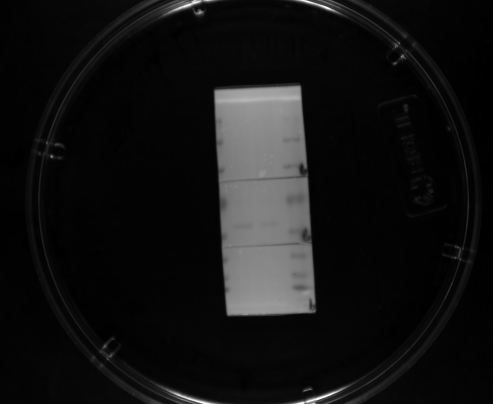


GAPDH


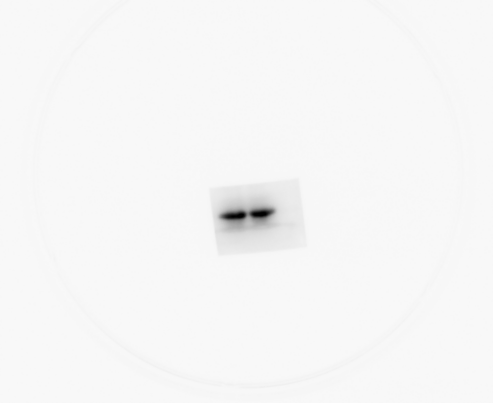


P-ATM


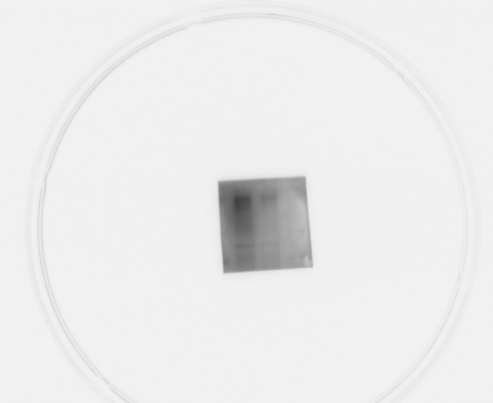


P-P53


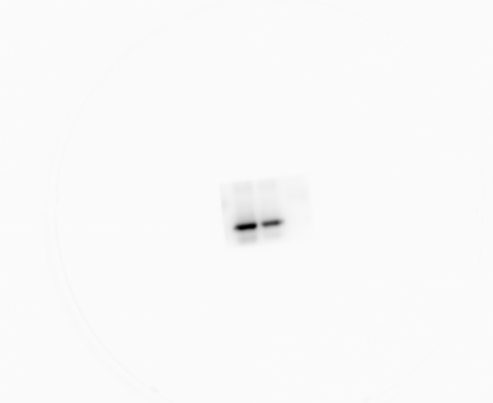


γH2AX


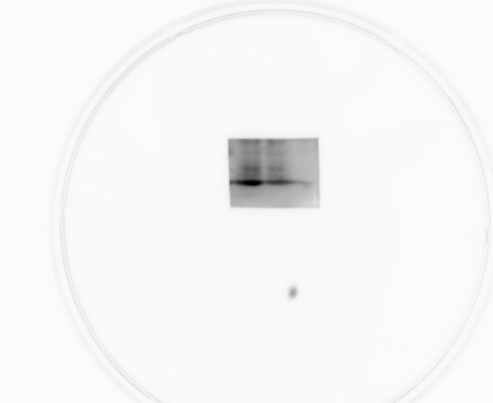


# fig6I

## DMS114


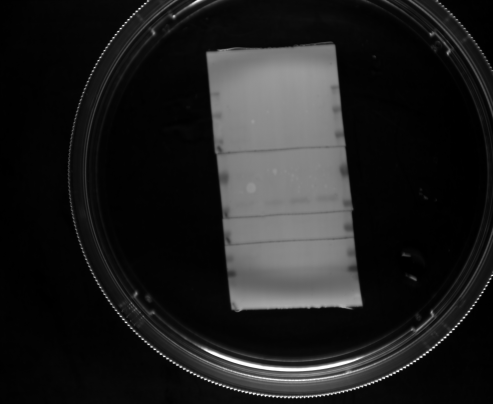


GAPDH


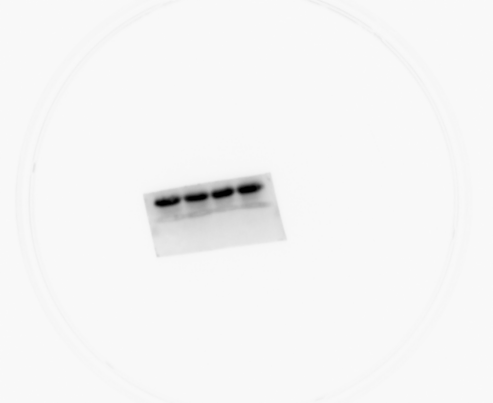


HINT1


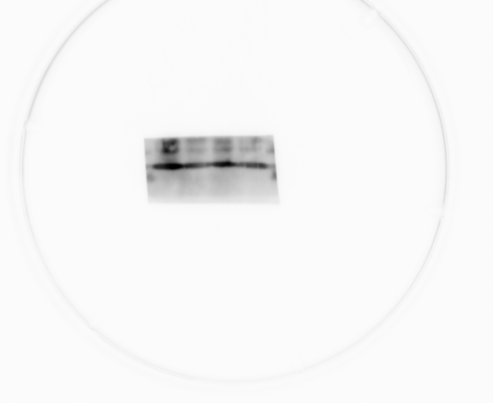


P-ATM


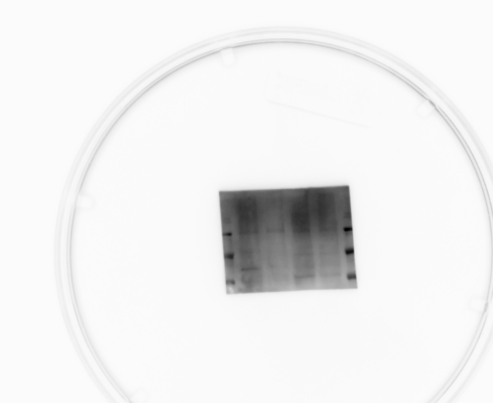


P-P53


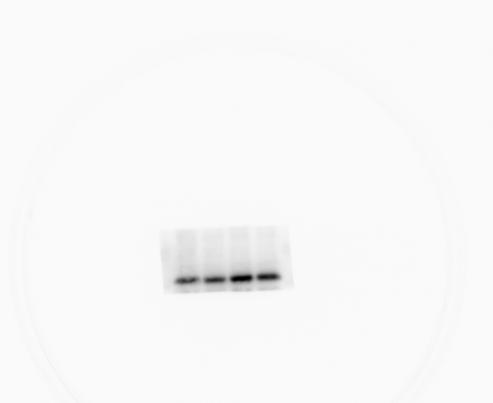


γH2AX


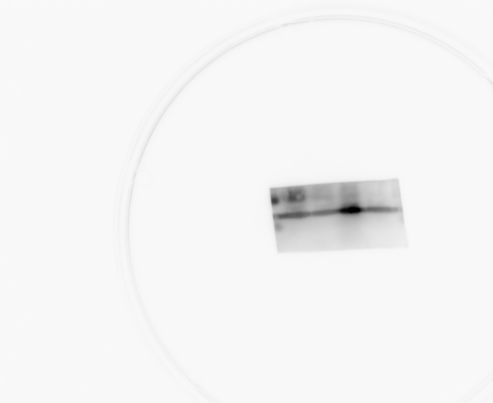


## H446


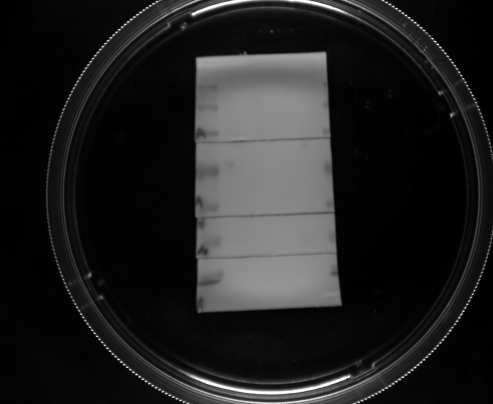


GAPDH


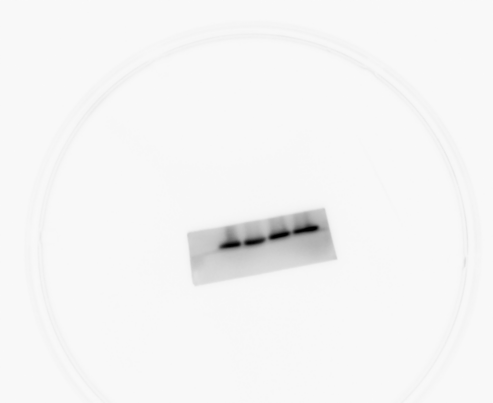


HINT1


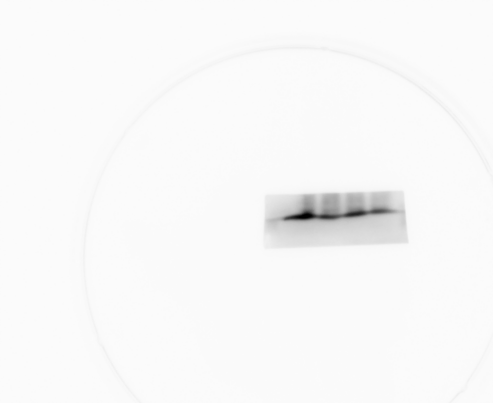


P-ATM


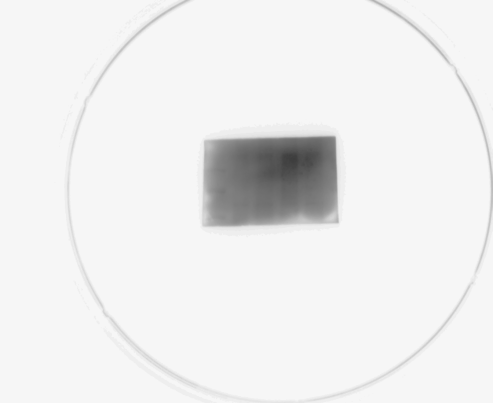


P-P53


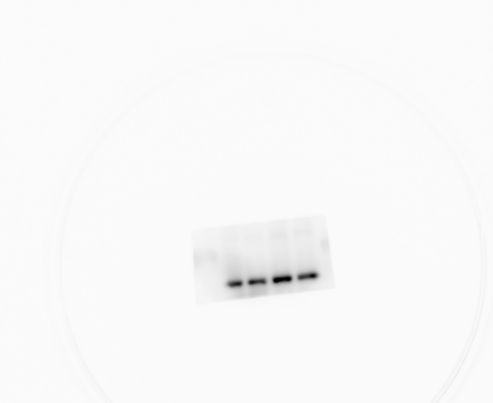


γH2AX


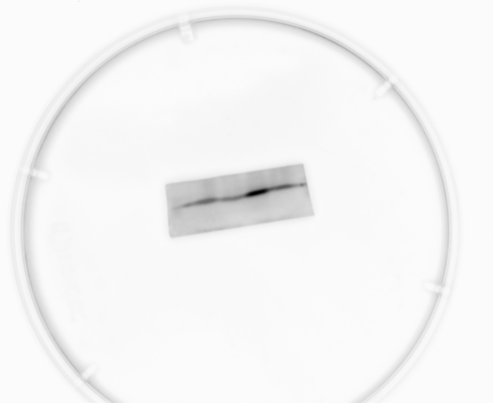


# fig6J

## DMS114-DDP


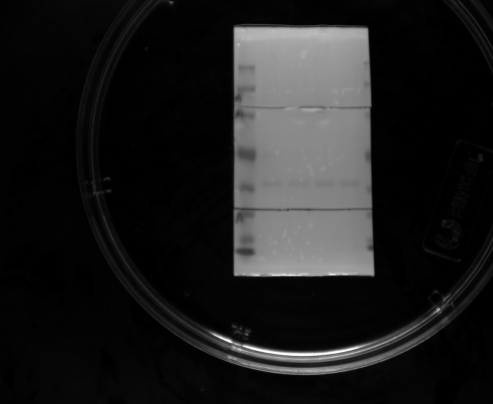


GAPDH


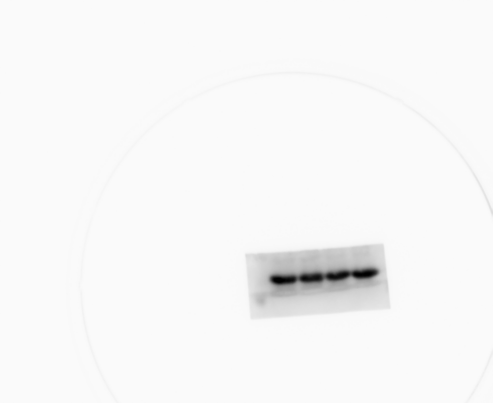


HINT1


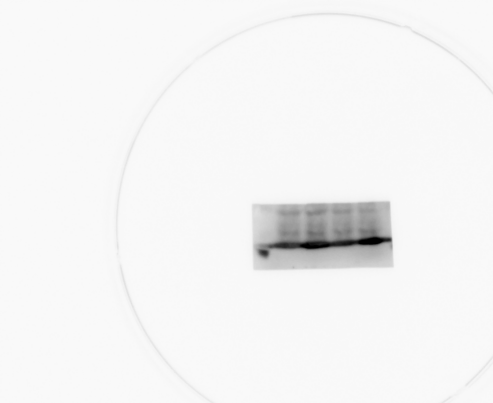


P-ATM


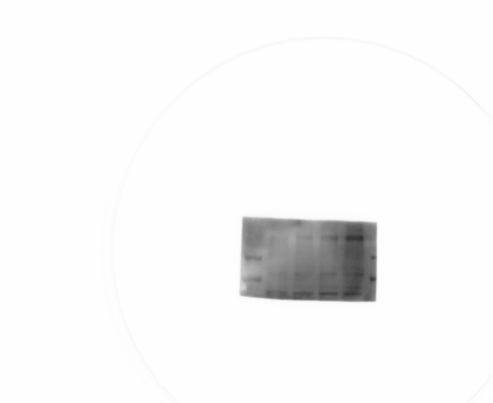


P-P53


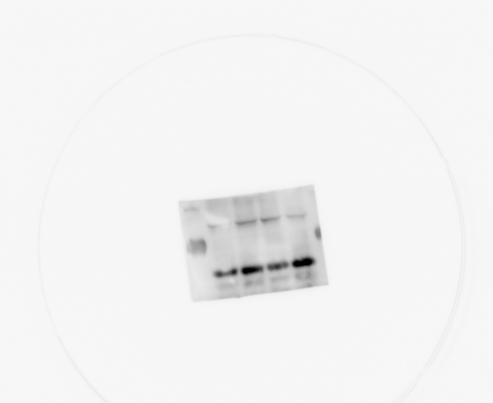


γH2AX


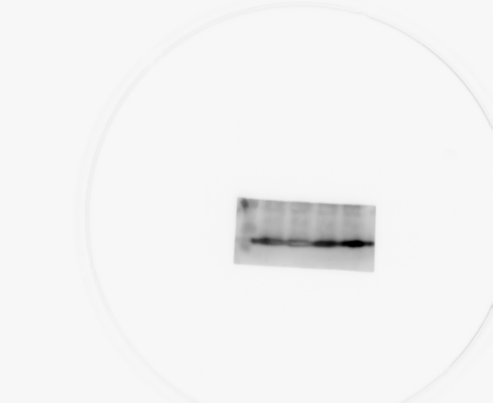


## H446-DDP


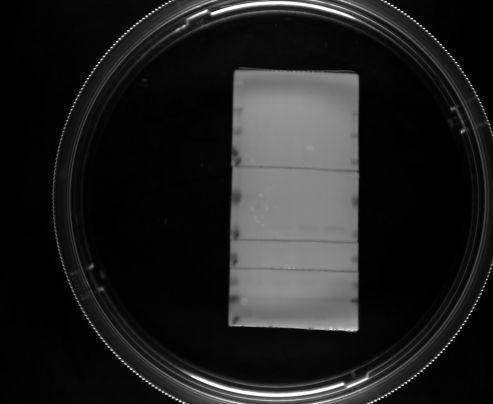


GAPDH


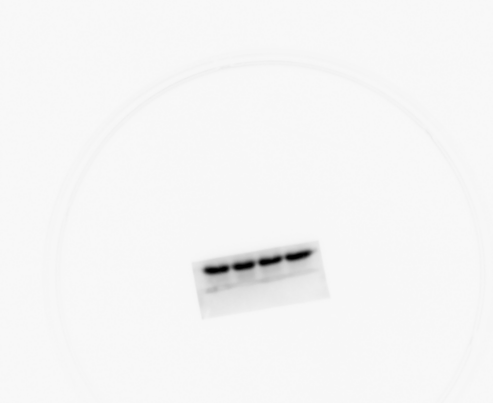


HINT1

P-ATM

P-P53

γH2AX

# fig6K

## DMS114

GAPDH

HINT1

P-ATM

P-P53

γH2AX

VANGL2

## H446

GAPDH

HINT1

P-ATM

P-P53

γH2AX

VANGL2

# fig6L

## DMS114-DDP

GAPDH

HINT1

P-ATM

P-P53

γH2AX

VANGL2

## H446-DDP

GAPDH

HINT1

P-ATM

P-P53

γH2AX

VANGL2
