# Supplementary material for: VANGL2 downregulates HINT1 to inhibit the ATM-p53 pathway and promote cisplatin resistance in small cell lung cancer
Source: Cell Death Discov. 2025 Apr 8;11:153. doi: 10.1038/s41420-025-02424-w (PMC11979007; doi:10.1038/s41420-025-02424-w)
Supplement: Supplementary file 2 — Supplementary Material [file 41420_2025_2424_MOESM2_ESM.docx]

**Table S1. Sequences of shRNA**

| shRNA | Target Seq |
| --- | --- |
| shVANGL2-1 | GGTCTCCTACTGGCTCTTCTA |
| shVANGL2-2 | GCTTCTACAACGTTGGCCATC |

**Table S2. Sequences of siRNA**

| siRNA | sense（5'-3'） | antisense（5'-3'） |
| --- | --- | --- |
| HINT1-1 | CUGAAUAAGGGUUAUCGAATT | UUCGAUAACCCUUAUUCAGTT |
| HINT1-2 | GAUACCCAAGAAACAUAUATT | \| UAUAUGUUUCUUGGGUAUCTT \| \| --- \| |

**Table S3. Primers for real-time PCR**

| mRNA | Forward primer (5'-3') | Reverse primer (5'-3') |
| --- | --- | --- |
| VANGL2 | ACTCGGGCTATTCCTACAAGT | CTCGACTCTTAGAGCGGTGTC |
| HINT1 | TTCCCCTCAAGCACCAACAC | GCCCAGATCAGCAGCACATT |
| GAPDH | GGAGCGAGATCCCTCCAAAAT | \| GGCTGTTGTCATACTTCTCATGG \| \| --- \| |

**Table S4. Antibody Information**

| **Antibodies** | **SOURCE** | **Catalog Number** |
| --- | --- | --- |
| VANGL2 | Proteintech | Cat#: 21492-1-AP |
| HINT1 | Abmart | Cat#: [MU129079](http://www.ab-mart.com.cn/page.aspx?node= 77 &id= 99247" \t "https://www.ab-mart.com.cn/_blank) |
| BCL-2 | Bioss | Cat#: bs-0032R |
| BAX | Bioss | Cat#: bs-4564R |
| GAPDH | Bioworld | Cat#: ap0063 |
| HSP90 | Proteintech | Cat#: 60318-1-Ig |
| [Phospho-p53(Ser46](http://www.ab-mart.com.cn/page.aspx?node= 77 &id= 1218" \t "https://www.ab-mart.com.cn/_blank)) | Abmart | Cat#: [PS06218](http://www.ab-mart.com.cn/page.aspx?node= 77 &id= 120967" \t "https://www.ab-mart.com.cn/_blank) |
| Phospho-ATM(Ser1981) | Santa Cruz | Cat#: sc-47739 |
| γH2AX | Origene | Cat#: TA346960 |
| FLAG | MilliporeSigma | Cat#: F1804 |
| IgG | Bioss | Cat#: bs-0296PC |


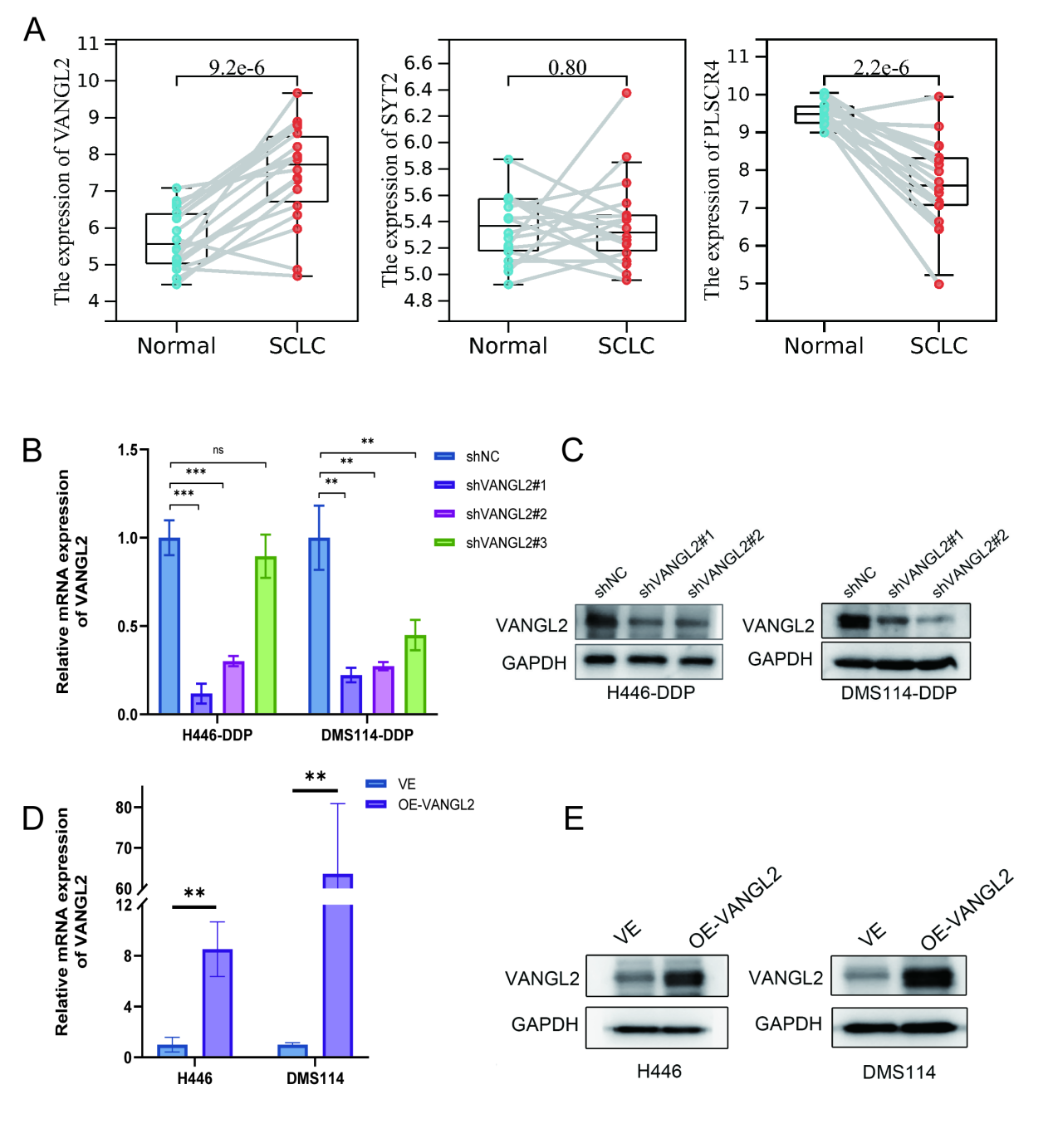


Figure S1. (A)The mRNA expression levels of VANGL2, SYT2 and PLSCR4 between SCLC and adjacent normal tissues. (B)The qRT-PCR assays examined the down-regulation of VANGL2 mRNA levels in H446-DDP and DMS114-DDP cells. **P<0.01; ***P<0.001; ns, not significant. (C)Western blot assays examined the down-regulation of VANGL2 protein levels in H446-DDP and DMS114-DDP cells. (D)The qRT-PCR assays examined the overexpression of VANGL2 mRNA levels in H446 and DMS114 cells. **P<0.01. (E)Western blot assays examined the overexpression of VANGL2 protein levels in H446 and DMS114 cells.


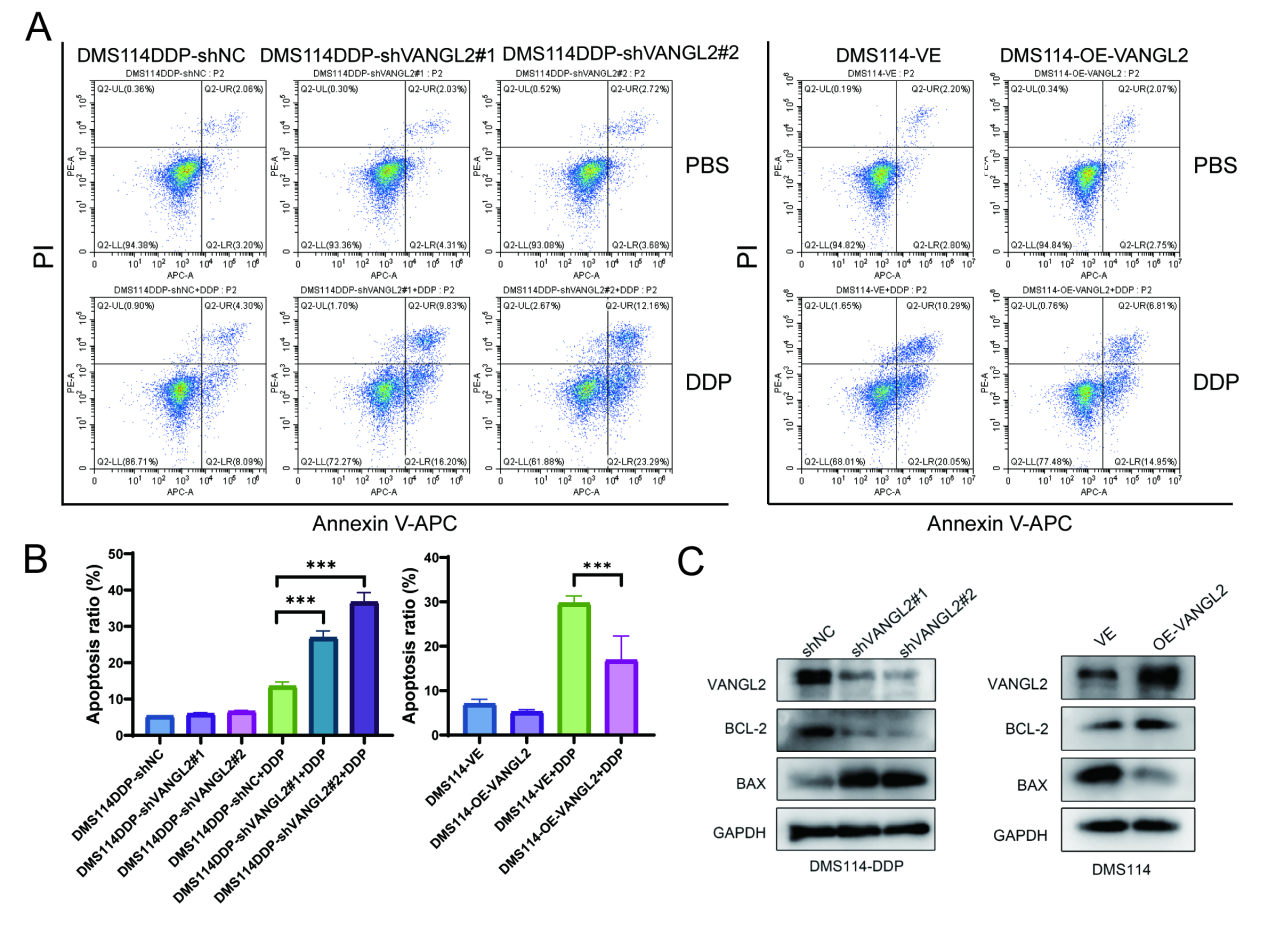


Figure S2.(A-B)Downregulation of VANGL2 expression in DMS114-DDP resulted in the increase in cisplatin-induced apoptosis. Overexpression of VANGL2 in DMS114 resulted in the reduction of cisplatin-induced apoptosis. ***P<0.001. (C)Apoptosis-related protein levels were measured by Western blot assays after treatment with cisplatin in VANGL2 knockdown or VANGL2 overexpressing cells.


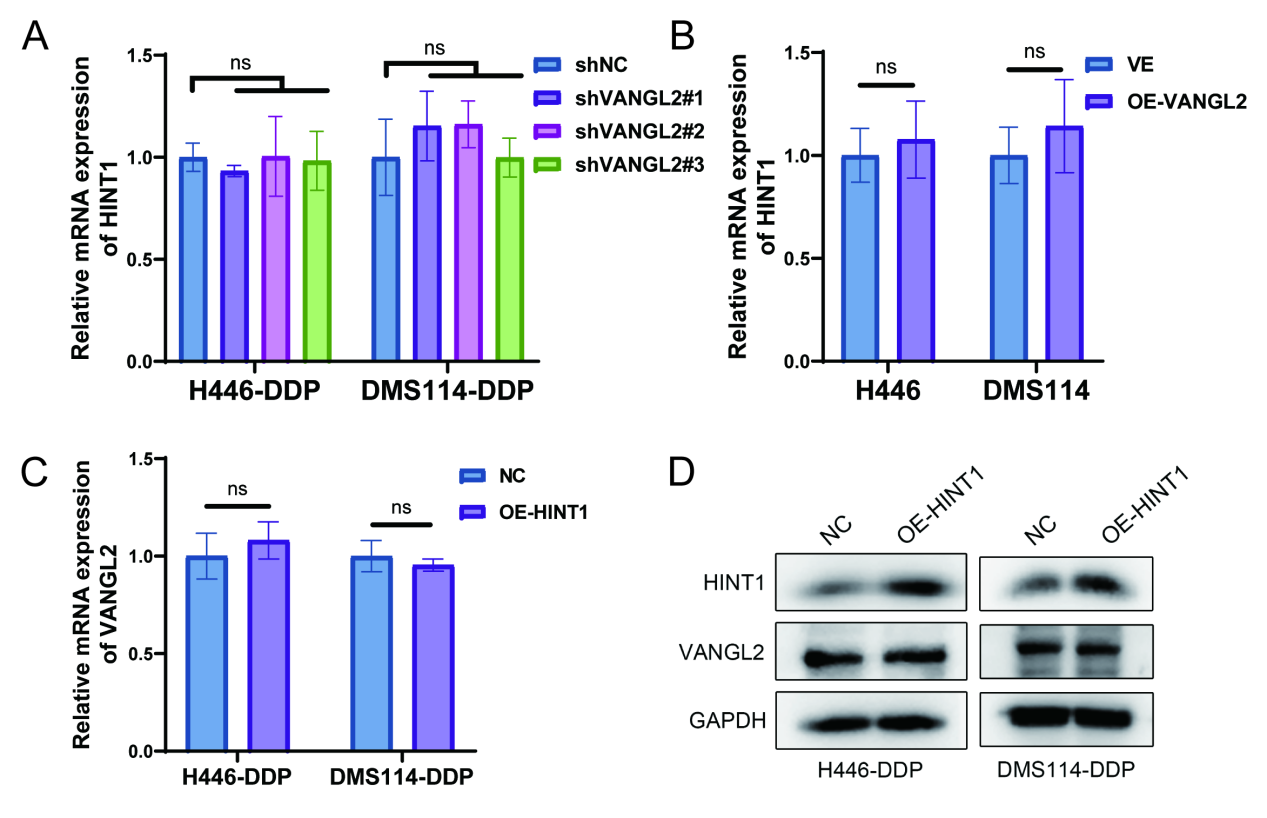


Figure S3. (A)The qRT-PCR detected the mRNA of HINT1 after knockdown of VANGL2. ns, not significant. (B)The qRT-PCR detected the mRNA of HINT1 after overexpression of VANGL2. ns, not significant. (C)The qRT-PCR detected the mRNA of VANGL2 after overexpression of HINT1 expression. ns, not significant. (D)The Western blot assays detected the protein levels of VANGL2 after overexpression of HINT1 expression.

**
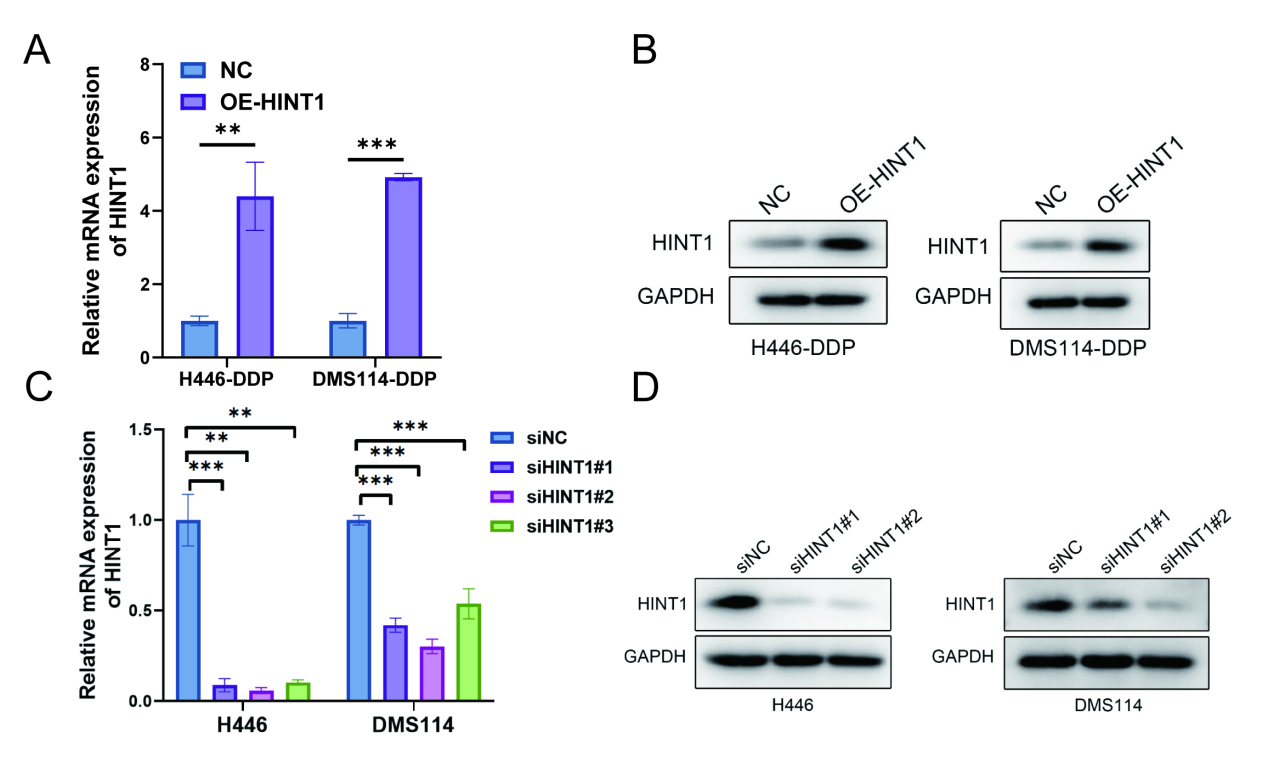
**

Figure S4.(A)The qRT-PCR assays detected the overexpression of HINT1 mRNA expression levels in H446-DDP and DMS114-DDP cells. **P<0.01; ***, P<0.001. (B) The Western blot assays detected the overexpression of HINT1 protein expression levels in H446-DDP and DMS114-DDP cells. (C)The qRT-PCR detected the down-regulation of HINT1 mRNA in H446 and DMS114 cells. (D)The Western blot assays detected the down-regulation of HINT1 protein expression levels in H446 and DMS114 cells.


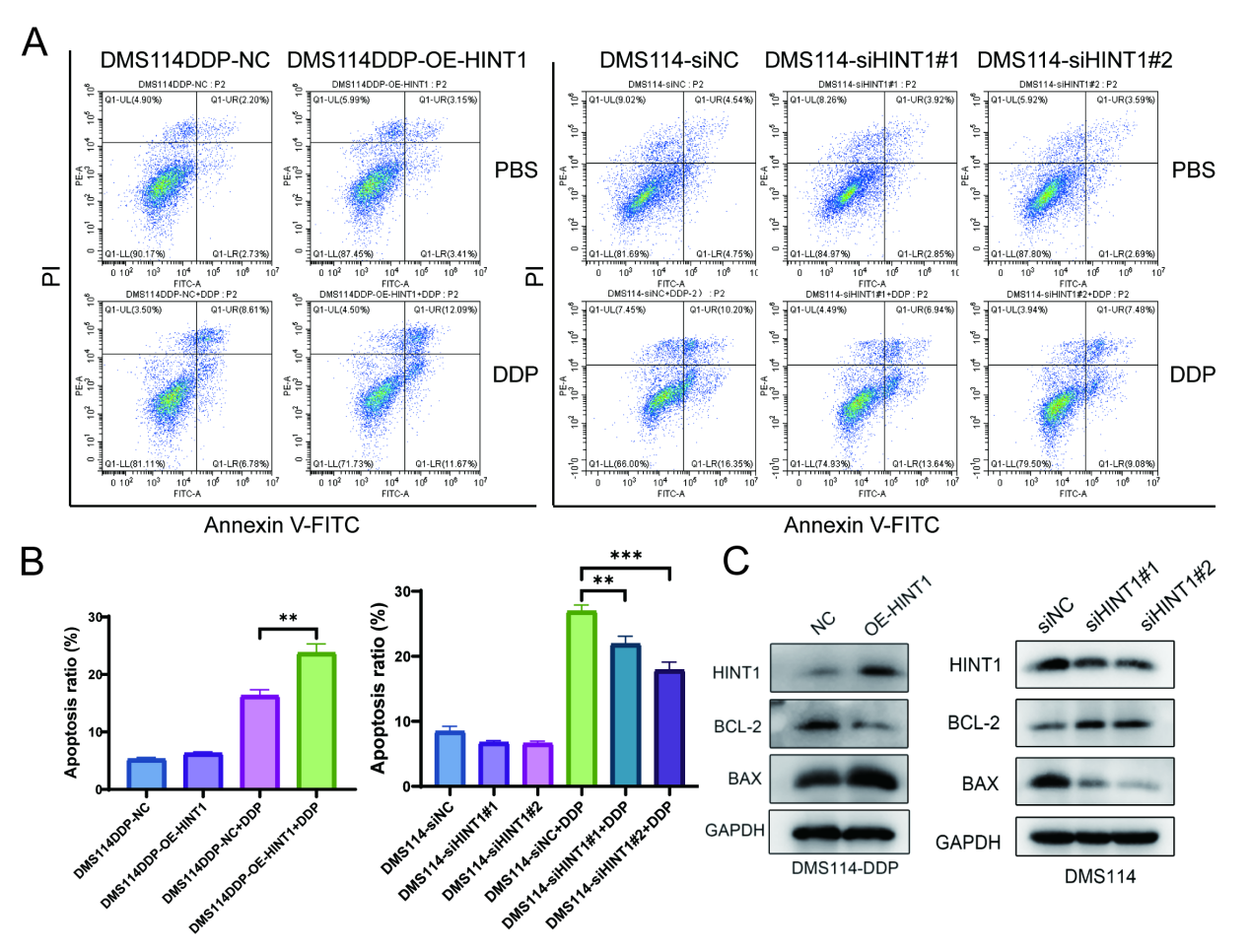


Figure S5.(A-B)Overexpression of HINT1 expression in DMS114-DDP resulted in the increase in cisplatin-induced apoptosis. Downregulation of HINT1 in DMS114 resulted in the reduction of cisplatin-induced apoptosis. **P<0.01; ***P<0.001. (C)Apoptosis-related protein levels were measured by Western blot assays in HINT1-overexpressing or HINT1-knockdown cells following treatment with cisplatin.

**
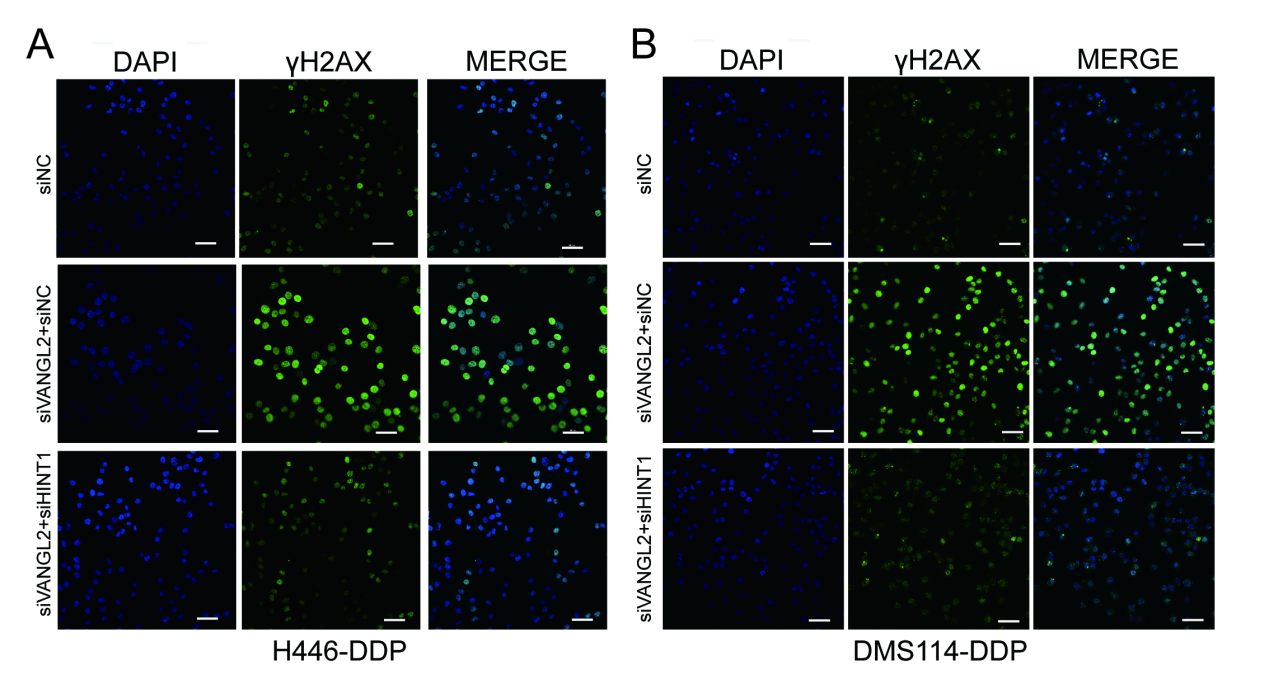
**

Figure S6. (A-B)Immunofluorescence assays showed γH2AX expression in SCLC cisplatin-resistant cells treated with cisplatin for 24 hours, following silencing with siNC, si-VANGL2, or both si-VANGL2 and si-HINT1. Scale bar = 50µm.
